# Synergistic stabilization by nitrosoglutathione-induced thiol modifications in the stromal interaction molecule-2 luminal domain suppresses basal and store operated calcium entry

**DOI:** 10.1038/s41598-020-66961-3

**Published:** 2020-06-23

**Authors:** Matthew J. Novello, Jinhui Zhu, MengQi Zhang, Qingping Feng, Peter B. Stathopulos

**Affiliations:** 1 Department of Physiology and Pharmacology, Schulich School of Medicine and Dentistry, the University of Western Ontario, London, Ontario, N6A5C1 Canada; 2 Present Address: Dentistry, Schulich School of Medicine and Dentistry, the University of Western Ontario, London, Ontario, N6A5C1 Canada; 3 Present Address: Faculty of Medicine, University of Ottawa, Ottawa, Ontario, K1H8M5 Canada

**Keywords:** Structural biology, Calcium signalling, Post-translational modifications

## Abstract

Stromal interaction molecule−1 and −2 (STIM1/2) are endoplasmic reticulum (ER) membrane-inserted calcium (Ca^2+^) sensing proteins that, together with Orai1-composed Ca^2+^ channels on the plasma membrane (PM), regulate intracellular Ca^2+^ levels. Recent evidence suggests that *S*-nitrosylation of the luminal STIM1 Cys residues inhibits store operated Ca^2+^ entry (SOCE). However, the effects of thiol modifications on STIM2 during nitrosative stress and their role in regulating basal Ca^2+^ levels remain unknown. Here, we demonstrate that the nitric oxide (NO) donor nitrosoglutathione (GSNO) thermodynamically stabilizes the STIM2 Ca^2+^ sensing region in a Cys-specific manner. We uncovered a remarkable synergism in this stabilization involving the three luminal Cys of STIM2, which is unique to this paralog. *S*-Nitrosylation causes structural perturbations that converge on the face of the EF-hand and sterile α motif (EF-SAM) domain, implicated in unfolding-coupled activation. In HEK293T cells, enhanced free basal cytosolic Ca^2+^ and SOCE mediated by STIM2 overexpression could be attenuated by GSNO or mutation of the modifiable Cys located in the luminal domain. Collectively, we identify the Cys residues within the N-terminal region of STIM2 as modifiable targets during nitrosative stress that can profoundly and cooperatively affect basal Ca^2+^ and SOCE regulation.

## Introduction

Stromal-interaction molecules (STIM)s are endoplasmic reticulum (ER) membrane-inserted calcium (Ca^2+^) sensors that respond to fluctuations in luminal stored Ca^2+^ levels^1,2^. In *Homo sapiens*, two STIM homologs exist: stromal interaction molecule−1 (STIM1) and −2 (STIM2)^3^. Upon ER luminal Ca^2+^ store depletion, human STIM1 undergoes structural changes that promote oligomerization and translocation to ER-plasma membrane (PM) junctions^4–6^. At these junctions, STIM1 interacts with Orai1 proteins, which are the pore-forming subunits of Ca^2+^ release activated Ca^2+^ (CRAC) channels^7–9^. The direct interaction of STIM1 with Orai1 facilitates gating of the CRAC channels to induce the influx of extracellular Ca^2+^ into the cytosol^10–12^, otherwise known as store operated Ca^2+^ entry (SOCE)^13,14^. The human STIM2 paralog similarly regulates SOCE; however, STIM2 is less efficient than STIM1 in this cellular process^15–18^. Instead, STIM2 is more intimately involved in the regulation of basal Ca^2+^ levels^15^. Interestingly, in lower order eukaryotes such as *Drosophila melanogaster* and *Caenorhabditis elegans*, only one *STIM* gene product has been identified^3^.

**Figure 1:**
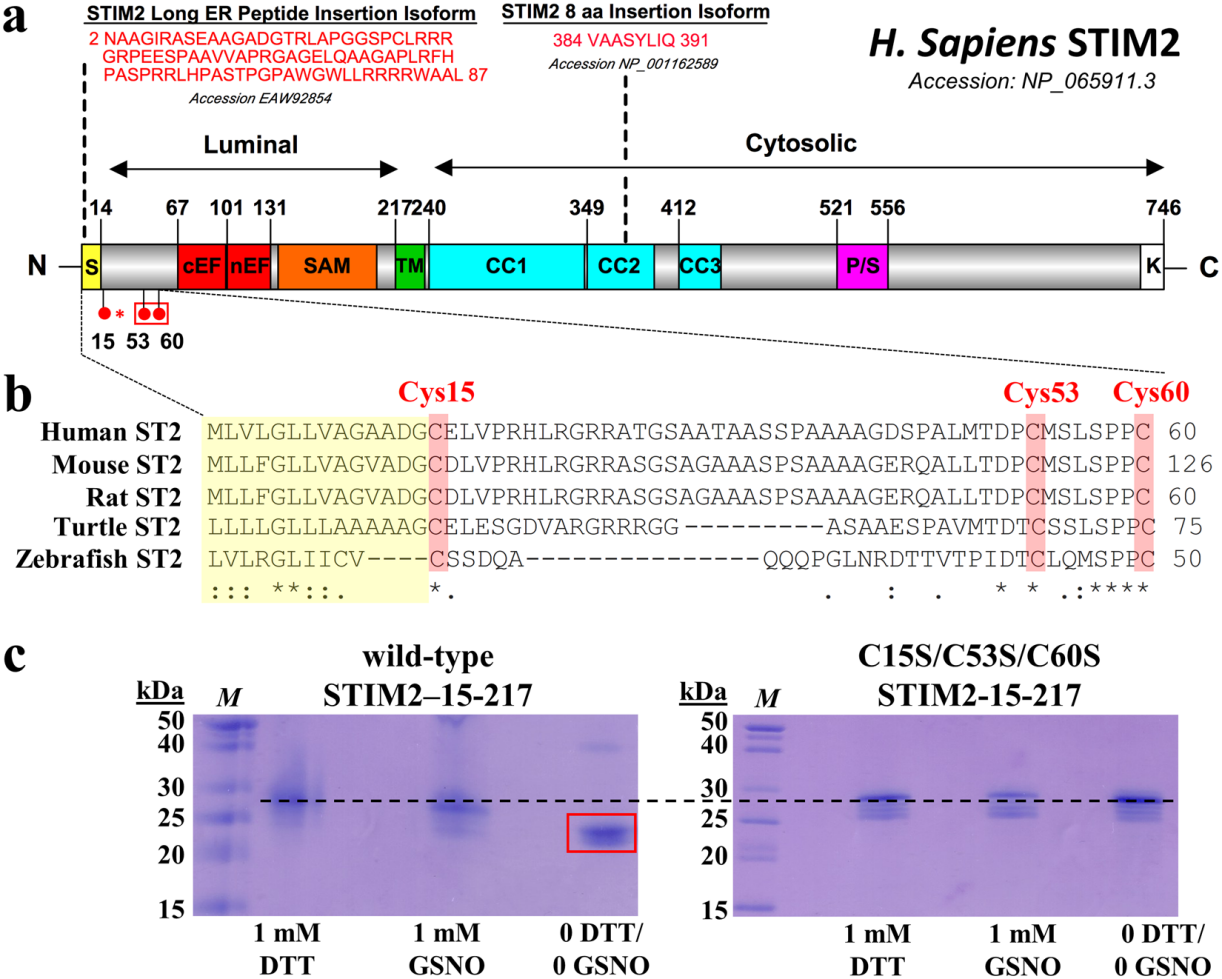
Domain architecture and sequence alignments of STIM2 proteins. (**a**) Key structural features on the human STIM2 domain architecture. Residue ranges are shown on top or bottom of the demarcated domains. Broken lines specify amino acid insertions associated with specific STIM2 isoforms. The position of the luminal Cys residues are shown as red spheres, and the conserved Cys residues between STIM homologs are encompassed in a red box. The Cys unique to STIM2 is emphasized by a red asterisk. The cellular localization the STIM regions is indicated on top of the diagrams. S, signal peptide; cEF, canonical EF-hand motif; nEF, non-canonical EF-hand motif; SAM, sterile α motif; TM, transmembrane; CC1, 2, 3, coiled-coil−1, −2, −3; P/S, proline-serine-rich region; K, polybasic region. **(b)** Multiple sequence alignment of the STIM2 variable N-terminal region, including *Homo sapiens* STIM2 (Human ST2; NCBI accession NP_065911.3); *Mus musculus* STIM2 (Mouse ST2; NCBI accession AAI45002.1); *Rattus norvegicus* STIM2 (Rat ST2; NCBI accession NP_001099220); *Terrapene exicana triunguis* STIM2 (Turtle ST2; NCBI accession XP_024060054.1); and *Danio rerio* STIM2 (Zebrafish ST2; NCBI accession XP_002660454.3). Fully conserved Cys residues between STIM2 homologs are shaded in red. ER signal peptide regions are shaded in yellow. Alignments were performed using Clustal Omega^55^. Fully conserved (*); highly conserved (:); partially conserved (.). **(c)** SDS-PAGE migration pattern of wild-type and Cys15Ser/Cys53Ser/Cys60Ser STIM2 15–217 under *S*-nitrosylating, reducing and oxidizing conditions. Protein samples (0.25 mg mL^−1^) were separated on a 12.5% (w/v) SDS-PAGE gel and visualized by Coomassie blue staining. Samples were not boiled and separated by empty lanes due to the labile nature of *S*-nitrosylation. STIM2 15–217 exhibits a delayed migration relative to the theoretical monomer molecular weight of ~24 kDa, as previously observed with STIM1 EF-SAM^19,20^. *M* denotes molecular weight Marker. The dashed line is shown as a horizontal reference. The red box indicates the increased mobility due to intramolecular disulfide-bond formation. Gels are representative of n = 2 separate experiments.

The highly conserved EF-hand and sterile α-motif (EF-SAM) domains of STIMs are the core luminal protein machinery that sense ER Ca^2+^ changes^17,19,20^, while the cytosolic STIM-Orai1-activating-region (SOAR) coiled-coils are the highly conserved cytosolic domains that couple to and open Orai1 channels^11,12^. Compared to STIM1, the STIM2 EF-SAM domain has a higher stability that suppresses the extent and rate of oligomerization^17^, and the SOAR domain binds to Orai1 with lower affinity^16^, making STIM2 less efficient at mediating SOCE. Nevertheless, several structural and mechanistic differences between the STIM homologs allow STIM2 to more effectively regulate basal Ca^2+^ (Fig. 1a). First, the Ca^2+^-sensing EF-hand motif of human STIM2 has an apparently lower Ca^2+^ affinity [equilibrium dissociation constant (K_d_) ~0.4–0.8 mM]^15,17,21^ compared to STIM1 (K_d_ ~0.2–0.6 mM), which more closely matches resting ER Ca^2+^ levels of ~0.4–0.7 mM^19^. Consequently, STIM2 becomes activated after smaller decreases in luminal Ca^2+^ levels, and a higher proportion of STIM2 molecules are active at resting ER Ca^2+^ levels^15^. Second, the C-terminal polybasic domain of STIM2 has a stronger affinity for PM phosphatidyl 4,5-bisphosphate (PIP_2_) compared to STIM1, which is necessary for STIM2 ER-PM localization^12,22,23^. Third, compared to STIM1 SOAR^11,12^, STIM2 SOAR is less sequestered, placing the coiled-coil domains that interact with Orai1 closer to the PM^24^. Thus, in addition to mediating SOCE, STIM2 also functions as an efficient regulator of basal Ca^2+^.

Post-translational modifications can profoundly impact the structure and function of STIMs^25^. For example, oxidative stress causes *S*-glutathionylation within the STIM1 luminal domain, leading to constitutive Ca^2+^ entry^26^. Additionally, we recently demonstrated that two Cys residues within the variable N-terminal region of STIM1 (Cys49 and Cys56) can undergo *S*-nitrosylation, stabilize the luminal domain of STIM1 and inhibit SOCE^27,28^. While these two Cys residues are conserved between STIM vertebrate homologs (Cys53 and Cys60 in STIM2), STIM2 contains an additional Cys, non-existent in STIM1, within its variable N-terminal region (Cys15)^29^, which may serve as an added regulatory input site after thiol modifications (Fig. 1a). Interestingly, this unique Cys is present in the earliest known *STIM2*-containing organism (*Danio rerio*; zebrafish)^3^, highlighting the functional importance of this residue (Fig. 1b). Nevertheless, the susceptibility of any STIM2 luminal Cys to post-translational modifications and the subsequent effects on the Ca^2+^ sensing mechanism are unknown. Therefore, the goal of this study was to determine if the STIM2 luminal Cys residues can be *S*-nitrosylated, the biophysical and structural consequences of these modifications and how Cys15, which is unique to STIM2, contributes to structural, mechanistic and functional disparities between the homologs.

Here, we show that the luminal domain of STIM2 is indeed sensitive to *S*-nitrosylation by the nitric oxide donor nitrosoglutathione (GSNO), enhancing the stability, perturbing the structure and inhibiting the Ca^2+^-regulating function of STIM2. Our data suggest that all three Cys residues within the variable N-terminal region of STIM2 (Cys15, Cys53, and Cys60) can be modified by GSNO. *S*-Nitrosylation of these residues thermally and thermodynamically stabilizes the luminal domain via altered interactions of the variable N-terminal region with an isolated face of the Ca^2+^-sensing EF-SAM core. Remarkably, substitution of the unique Cys (Cys15Ser) attenuated *S*-nitrosylation-induced stabilization. Congruently, promoting *S*-nitrosylation in HEK293T cells significantly decreased resting cytosolic Ca^2+^ levels and SOCE. Unexpectedly, mutating the conserved Cys residues in the luminal region resulted in a loss of STIM2 function, underscoring the sensitivity of the protein to variations at these Cys positions. Collectively, we identify the Cys residues within the N-terminal region of STIM2 as modifiable targets during nitrosative stress that can profoundly affect the Ca^2+^ sensing function of the molecule in the regulation of basal cytosolic Ca^2+^ and SOCE.

## Results

### GSNO stabilizes human STIM2 15–217 under Ca^2+^ deplete and replete conditions

The unbinding of Ca^2+^ from the EF-hand concomitant with EF-SAM structural destabilization are hallmarks of STIM mediated SOCE initiation^17,19,20^. Recent evidence indicates that *S*-nitrosylation of the variable N-terminal region of human STIM1 enhances the stability of the full luminal domain and inhibits SOCE^27,28^. Therefore, we sought to determine whether *S*-nitrosylation also affects the stability of the human STIM2 luminal domain. Using a pET-28a vector and *Escherichia coli*, we expressed and purified the full human STIM2 luminal domain corresponding to residues 15–217 (NCBI accession number NP_065911.3; STIM2 15–217), which contains three modifiable Cys. *S*-Nitrosylation of these Cys was promoted by exchange from a dithiothreitol (DTT)-containing buffer to a GSNO-containing buffer using ultrafiltration. The ability of this approach to efficiently promote GSNO-mediated modification of the STIM1 luminal Cys residues was previously confirmed by solution nuclear magnetic resonance (NMR) spectroscopy^28^. Here, we assessed the migration of STIM2 15–217 by SDS-PAGE to verify that *S*-nitrosylation rather than disulfide bond formation was favoured using this same protocol. Ultrafiltration exchange of STIM2 15–217 into a buffer containing neither DTT nor GSNO resulted in enhanced migration on SDS-PAGE gels compared to the same protein in a DTT-containing buffer, indicative of intramolecular disulfide bond formation. In contrast, exchange into a GSNO-containing buffer minimally altered migration, consistent with the small mass changes caused by *S*-nitrosylation (Fig. 1c). We also note that this approach of maintaining excess NO donor to favour *S*-nitrosylation has been used in several other independent studies^30–33^.

**Figure 2:**
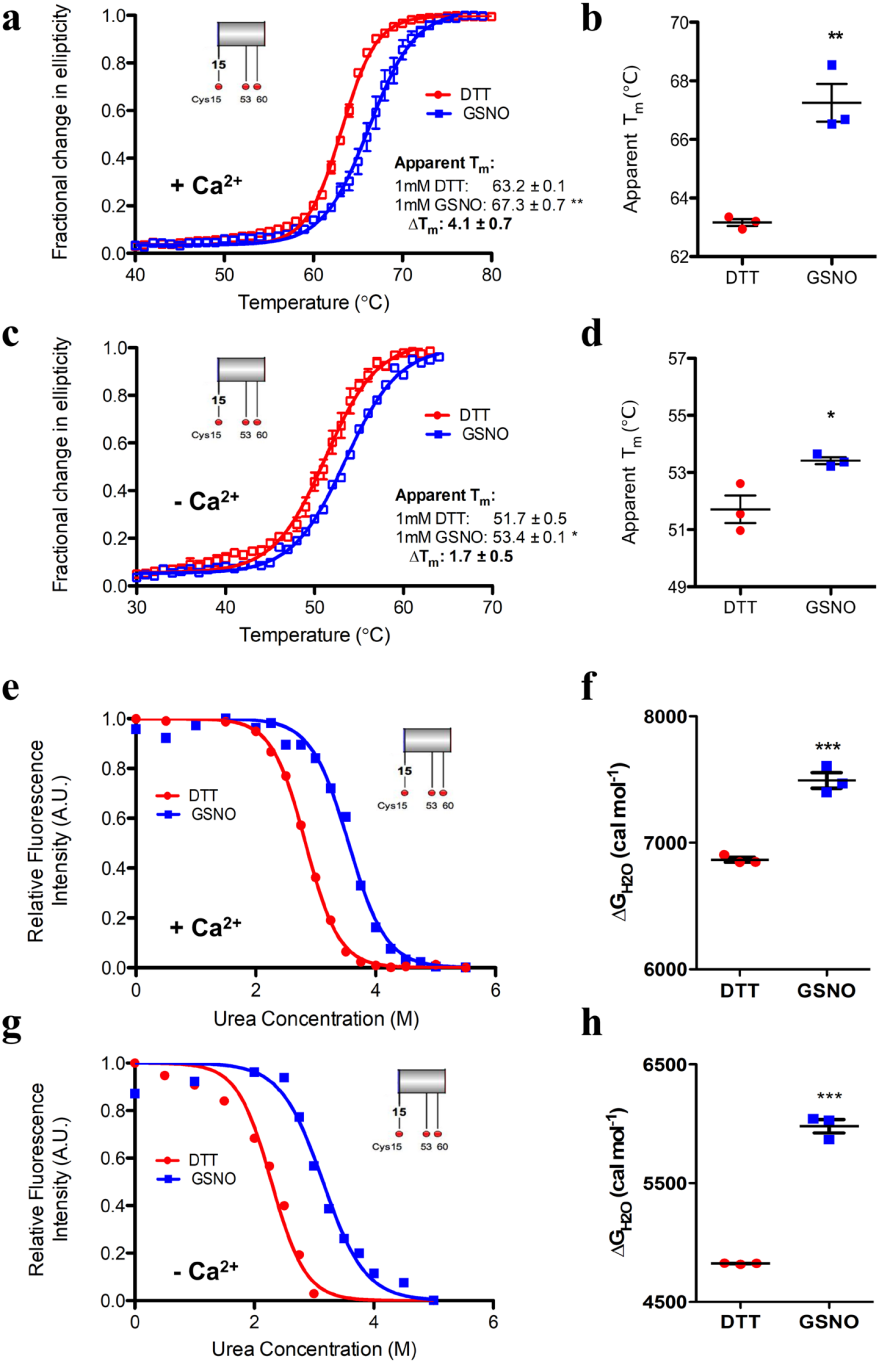
Thermal and thermodynamic stability of wild-type STIM2 15–217 under reducing and *S*-nitrosylating conditions. **(a)** Thermal melts of Ca^2+^-loaded (+Ca^2+^) wild-type STIM2 15–217 acquired in the presence of DTT or GSNO. **(b)** Comparison of apparent T_m_ values extracted from the +Ca^2+^ thermal melts. **(c)** Thermal melts of Ca^2+^-depleted (−Ca^2+^) wild-type STIM2 15–217 acquired in the presence of DTT or GSNO. **(d)** Comparison of apparent T_m_ values extracted from the −Ca^2+^ thermal melts. **(e)** Representative equilibrium denaturation curve of Ca^2+^-loaded wild-type STIM2 15–217 acquired in the presence of DTT or GSNO. **(f)** Comparison of Δ${{\rm{G}}}_{{{\rm{H}}}_{2}{\rm{O}}}$ extracted from the +Ca^2+^ denaturation curves. **(g)** Representative equilibrium denaturation curve of Ca^2+^-depleted wild-type STIM2 15–217 acquired in the presence of DTT or GSNO. **(h)** Comparison of Δ${{\rm{G}}}_{{{\rm{H}}}_{2}{\rm{O}}}$ extracted from the −Ca^2+^ denaturation curves. In (*a* – *d*), data were acquired with 0.5 mg mL^−1^ protein. In (*e* – *h*), data were acquired with 5 μM (*i.e*. 0.11 mg mL^−1^) protein at 20 °C. In (*a* – *h*), data acquired in the presence of 1 mM DTT and 1 mM GSNO are shown as red and blue datasets, respectively, and Ca^2+^-loaded samples contained 5 mM CaCl_2_. In (*a*, *c*, *e*, and *g*), insets show relative locations of modifiable Cys (red spheres). Data in (*b*, *d*, *f* and *h*) are reported as means ± SEM of n = 3 separate experiments for each group and were compared using an unpaired Student’s *t*-test. (*p < 0.05, **p < 0.01, ***p < 0.001 compared to the DTT control).

Having determined that our exchange protocol favours *S*-nitrosylation, we next gauged the thermal stability of STIM2 15–217 by monitoring the change in far-UV circular dichroism (CD) signal at 225 nm as a function of temperature. Indeed, excess GSNO significantly increased the apparent midpoint of temperature denaturation (T_m_) of Ca^2+^ loaded wild-type STIM2 15–217 by ~+4.1 °C compared to the control thermal melts acquired in the presence of 1 mM DTT, indicating a pronounced stabilization (Fig. 2a,b; Table 1). *S*-Nitrosylation induced a more modest increase of ~+1.7 °C in apparent T_m_ of Ca^2+^ depleted wild-type STIM2 15–217 (Fig. 2c,d; Table 1).

**Table 1:** Thermal stability parameters for luminal domain STIM protein with or without Ca^2+^ and in the absence or presence of GSNO.

	Apparent T_m_ (°C)^a^		
	−GSNO (+DTT)^b^	+GSNO^c^	ΔT_m_^d^
**STIM2 15–217**			
*Ca*^2+^ *loaded*			
Wildtype	63.2 ± 0.1	67.3 ± 0.7	**+4.1 ± 0.7**
C15S	63.4 ± 0.1	65.8 ± 0.5	**+2.4 ± 0.5**
C53S	63.6 ± 0.1	62.6 ± 0.2	**−1.0 ± 0.2**
C60S	62.0 ± 0.1	61.0 ± 0.2	**−1.0 ± 0.2**
C53S/C60S	60.0 ± 0.1	60.1 ± 0.1	**+0.1 ± 0.1**
C15S/C53S/C60S	60.8 ± 0.1	61.2 ± 0.1	+0.4 ± 0.1
*Ca*^2+^ *depleted*			
Wildtype	51.7 ± 0.5	53.4 ± 0.1	**+1.7 ± 0.5**
**STIM1 23–213**^**e**^			
*Ca*^2+^ *loaded*			
Wildtype	62.5 ± 0.5	64.3 ± 0.3	**+1.8 ± 0.6**
*Ca*^2+^ *depleted*			
Wildtype	41.7 ± 0.6	45.1 ± 0.9	**+3.4 ± 1.1**
C49S/C56S	37.9 ± 0.5	37.8 ± 0.4	**−0.1 ± 0.6**

^a^Apparent midpoints of temperature denaturation (T_m_).

^b^Data acquired in the presence of 1 mM DTT (*i.e*. reducing conditions).

^c^Data acquired in the presence of 1 mM GSNO (*i.e. S*-nitrosylating conditions).

^d^ΔT_m_ = T_m(+GSNO)_ − T_m(−GSNO)_; errors (±) propagated from ΔT_m,(−GSNO)_ and ΔT_m,(+GSNO)_ errors.

^e^Data is from reference^28^.

All data are mean ± SEM from n = 3 separate experiments for each group.

Due to the irreversibility of the thermal denaturation, which could distort unfolding profiles and influence the T_m_ readouts, we next conducted equilibrium chemical denaturation experiments to more reliably quantify and compare the *S*-nitrosylation-mediated changes in stability. Urea-induced denaturation of STIM2 15–217 monitored using changes in intrinsic protein fluorescence is completely reversible and thus amenable to equilibrium unfolding analyses. The change in Gibbs free energy of unfolding in water (Δ${{\rm{G}}}_{{{\rm{H}}}_{2}{\rm{O}}}$) and cooperativity of unfolding (*m*-value) were extracted from the chemical denaturation curves via the linear extrapolation method^34^ using a two state, folded to unfolded model. Consistent with the thermal melt data, our urea experiments showed that *S*-nitrosylation enhanced the thermodynamic stability of Ca^2+^ loaded wild-type STIM2 15–217 by +0.63 kcal mol^−1^ (Fig. 2e,f; Table 2). *S*-Nitrosylation induced a more pronounced stabilization for Ca^2+^-depleted STIM2 15–217 of +1.16 kcal mol^−1^ (Fig. 2g,h; Table 2).

**Table 2:** Thermodynamic stability parameters for luminal domain STIM protein with or without Ca^2+^ and in the absence or presence of GSNO.

	−GSNO (+DTT)^a^			+GSNO^b^			ΔΔ${{\rm{G}}}_{{{\rm{H}}}_{2}{\rm{O}}}$^f^ (kcal mol^−1^)
	Δ${{\rm{G}}}_{{{\rm{H}}}_{2}{\rm{O}}}$^c^ (kcal mol^−1^)	Cmid^d^ (M)	m-value^e^ (kcal mol^−1^ M^−1^)	Δ${{\rm{G}}}_{{{\rm{H}}}_{2}{\rm{O}}}$^c^ (kcal mol^−1^)	Cmid^d^ (M)	m-value^e^ (kcal mol^−1^ M^−1^)	
**STIM2 15–217**							
*Ca*^2+^ *loaded*							
Wildtype	6.87 ± 0.02	3.34 ± 0.01	2.05 ± 0.11	7.49 ± 0.06	3.58 ± 0.06	2.00 ± 0.17	+**0.62** ± **0.06**
*Ca*^2+^ *depleted*							
Wildtype	4.82 ± 0.02	2.85 ± 0.01	1.69 ± 0.08	5.98 ± 0.06	3.11 ± 0.03	1.92 ± 0.19	+**1.16** ± **0.06**
C15S	6.22 ± 0.02	3.47 ± 0.01	1.78 ± 0.12	6.71 ± 0.02	3.69 ± 0.03	1.82 ± 0.27	+**0.49** ± **0.03**
C53S/C60S	3.54 ± 0.01	2.36 ± 0.01	1.50 ± 0.07	3.63 ± 0.03	1.47 ± 0.08	2.48 ± 0.02	+**0.10** ± **0.03**
C15S/C53S/C60S	3.41 ± 0.17	2.39 ± 0.04	1.54 ± 0.17	3.40 ± 0.15	1.58 ± 0.11	2.38 ± 0.02	**−0.01** ± **0.23**
**STIM1 23–213**^**g**^							
*Ca*^2^^+^ *loaded*							
Wildtype	5.91 ± 0.03	3.09 ± 0.02	1.91 ± 0.07	7.87 ± 0.15	3.29 ± 0.06	2.39 ± 0.06	+**1.96** ± **0.15**
*Ca*^2^^+^ *depleted*							
Wildtype	1.73 ± 0.01	1.33 ± 0.01	1.30 ± 0.04	3.23 ± 0.06	1.76 ± 0.03	1.83 ± 0.06	+**1.50** ± **0.06**
C49S/C56S	1.75 ± 0.03	1.12 ± 0.02	1.55 ± 0.11	1.45 ± 0.06	1.15 ± 0.05	1.28 ± 0.07	**−0.30** ± **0.07**

^a^Data acquired in the presence of 1 mM DTT (*i.e*. reducing conditions).

^b^Data acquired in the presence of 1 mM GSNO (*i.e. S*-nitrosylating conditions).

^c^Gibbs free energy of unfolding in water; data fit to a two-state equilibrium unfolding model; globally fit to n = 3 separate experiments.

^d^Midpoint of chemical denaturation; calculated as Δ${{\rm{G}}}_{{{\rm{H}}}_{2}{\rm{O}}}$/*m*-value.

^e^Denaturant dependence of unfolding; globally fit to *n* = 3 separate experiments.

^f^ΔΔ${{\rm{G}}}_{{{\rm{H}}}_{2}{\rm{O}}}$ = Δ${{\rm{G}}}_{{{\rm{H}}}_{2}{\rm{O}}(+{\rm{GSNO}})}$ − Δ${{\rm{G}}}_{{{\rm{H}}}_{2}{\rm{O}}(-{\rm{GSNO}})}$_;_ errors (±) were propagated from Δ${{\rm{G}}}_{{{\rm{H}}}_{2}{\rm{O}}(-{\rm{GSNO}})}$ and Δ${{\rm{G}}}_{{{\rm{H}}}_{2}{\rm{O}}(+{\rm{GSNO}})}$ SEM.

^g^Data is from reference^27^.

All data are mean ± SEM from n = 3 separate denaturation curves.

Errors (±) in *m*-value are standard errors outputted from the global fits of *n* = 3 separate experiments for each group.

Collectively, our thermal and chemical denaturation assessments reveal that the full human STIM2 luminal domain is susceptible to *S*-nitrosylation, and this modification significantly enhances the stability of the domain under both activating (Ca^2+^-depleted) and deactivating (Ca^2+^-loaded) conditions.

### Cys15, Cys53 and Cys60 synergistically stabilize STIM2 15–217 in the presence of GSNO

*S*-Nitrosylation of both Cys49 and Cys56 in STIM1 is required to measurably stabilize the full luminal domain of STIM1^27^. STIM2 not only contains Cys residues at similar and conserved positions (Cys53 and Cys60), but also has an additional Cys residue at the N-terminus (Cys15), which we posit is also susceptible to *S*-nitrosylation. To determine whether the *S*-nitrosylation induced stabilization observed for STIM2 15–217 occurs in a Cys-specific manner, we created a series of Cys to Ser mutant STIM2 15–217 proteins. In total, five different mutant proteins were expressed and purified: Cys15Ser, Cys53Ser, Cys60Ser (single mutants), Cys53Ser/Cys60Ser (double mutant) and Cys15Ser/Cys53Ser/Cys60Ser (triple mutant). For all variants, protein expression levels and purities were similar to wild-type; however, the triple and double mutant proteins tended to aggregate and precipitate more readily compared to the other preparations.

**Figure 3:**
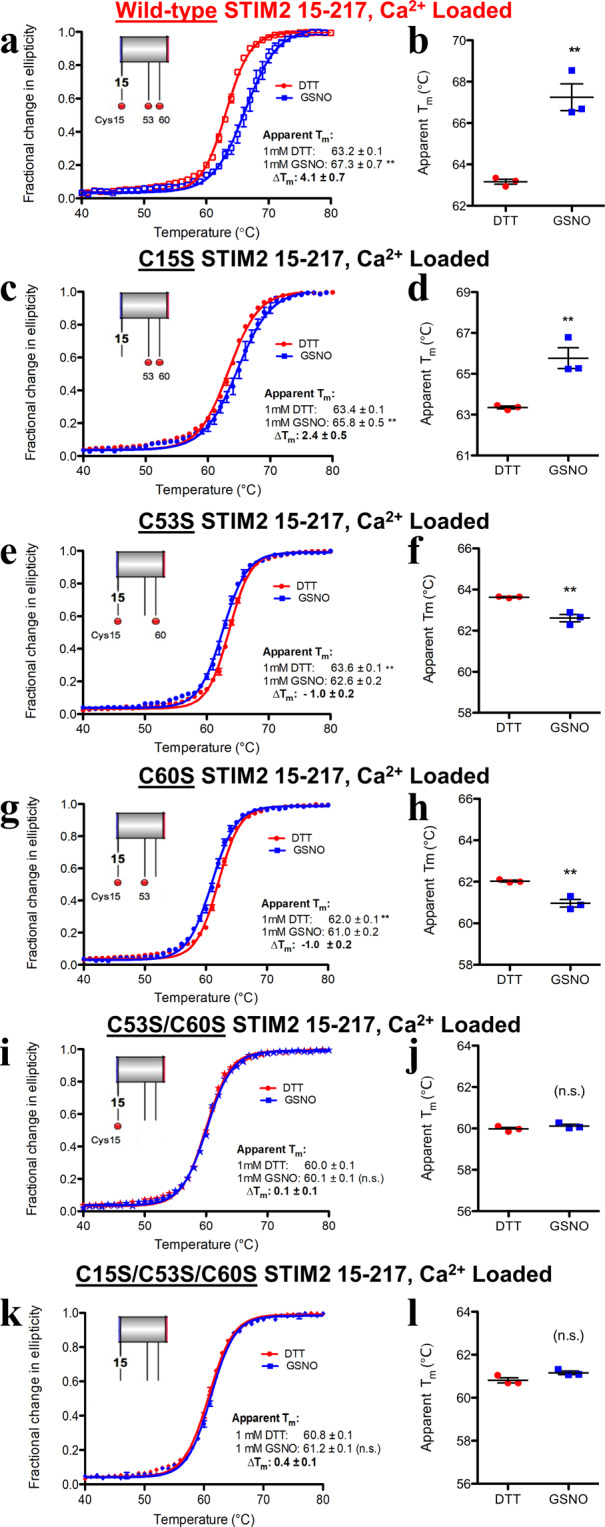
Thermal stability of Ca^2+^ loaded mutant STIM2 15–217 proteins under reducing and *S*-nitrosylating conditions. (**a, b**) Wild-type datasets for comparison (Reproduced from Fig. 2a,b). **(c)** Thermal melts of Ca^2+^-loaded (+Ca^2+^) Cys15Ser STIM2 15–217 acquired in the presence of DTT or GSNO. **(d)** Comparison of apparent T_m_ values extracted from the +Ca^2+^ Cys15Ser thermal melts. **(e)** Thermal melts of +Ca^2+^ Cys53Ser STIM2 15–217 acquired in the presence of DTT or GSNO. **(f)** Comparison of apparent T_m_ values extracted from the +Ca^2+^ Cys53Ser thermal melts. **(g)** Thermal melts of +Ca^2+^ Cys60Ser STIM2 15–217 acquired in the presence of DTT or GSNO. **(h)** Comparison of apparent T_m_ values extracted from the +Ca^2+^ Cys60Ser thermal melts. **(i)** Thermal melts of +Ca^2+^ Cys53Ser/Cys60Ser STIM2 15–217 acquired in the presence of DTT or GSNO. **(j)** Comparison of apparent T_m_ values extracted from the +Ca^2+^ Cys53Ser/Cys60Ser thermal melts. **(k)** Thermal melts of +Ca^2+^ Cys15Ser/Cys53Ser/Cys60Ser STIM2 15–217 acquired in the presence of DTT or GSNO. **(l)** Comparison of apparent T_m_ values extracted from the +Ca^2+^ Cys15Ser/Cys53Ser/Cys60Ser thermal melts. In (*a* – *l*), data were acquired with 0.5 mg mL^−1^ protein, 5 mM CaCl_2_ and either 1 mM DTT (red datasets) or 1 mM GSNO (blue datasets). In (*a*, *c*, *e*, *g, i* and *k*), insets show relative locations of modifiable Cys (red spheres). Data in (*b*, *d*, *f*, *h, j* and *l*) are reported as means ± SEM of n = 3 separate experiments for each group and were compared using an unpaired Student’s *t*-test. (**p < 0.01 and n.s. denotes not significant compared to the DTT control).

Under reducing conditions (*i.e*. 1 mM DTT) and in the presence of Ca^2+^, the Cys15Ser and Cys53Ser single mutant proteins marginally increased the T_m_ by ~+0.2 and ~+0.5 °C, respectively, compared to wild-type (Figs. 3a-f and S1). In contrast, the Cys60Ser mutation decreased the thermal stability by ~−1.1 °C compared to wild-type (Figs. 3a,b,g,h and S1). The Cys53Ser/Cys60Ser and Cys15Ser/Cys53Ser/Cys60Ser STIM2 15–217 luminal domains showed the largest decreases in T_m_ compared to wild-type by ~−3.2 and −2.3 °C, respectively (Figs. 3a,b,i-l and S1). Thus, despite the subtle sulfhydryl (Cys) to hydroxyl (Ser) variation, the STIM2 15–217 thermal stability is remarkably sensitive to changes at the three Cys positions.

We next evaluated the effect of GSNO on the stability of the variants retaining one or two modifiable Cys in the presence of Ca^2+^. GSNO caused a smaller increase in T_m_ of only ~+2.4 °C for the Cys15Ser mutant protein compared to wild-type (~+4.1 °C), indicating that the unique Cys15 of STIM2 15–217 plays a key role in the observed *S*-nitrosylation induced thermal stabilization (Fig. 3a-d**;** Table 1). In contrast, *S*-nitrosylation of the Cys53Ser and Cys60Ser single mutant proteins showed no apparent enhancement in stability, as GSNO significantly decreased the T_m_ values by ~−1.0 – −1.1 °C (Fig. 3e-h; Table 1). Both the Cys53Ser/Cys60Ser double mutant and Cys15Ser/Cys53Ser/Cys60Ser triple mutant thermal stabilities were insensitive to GSNO with the respective T_m_ values only marginally affected (Fig. 3i-l**;** Table 1). Thus, Cys15, which is unique to STIM2 orthologues, appears to only contribute to the GSNO-mediated stabilization of the luminal domain when Cys53 and Cys60, which are conserved among all vertebrate STIMs, are present and modifiable.

**Figure 4:**
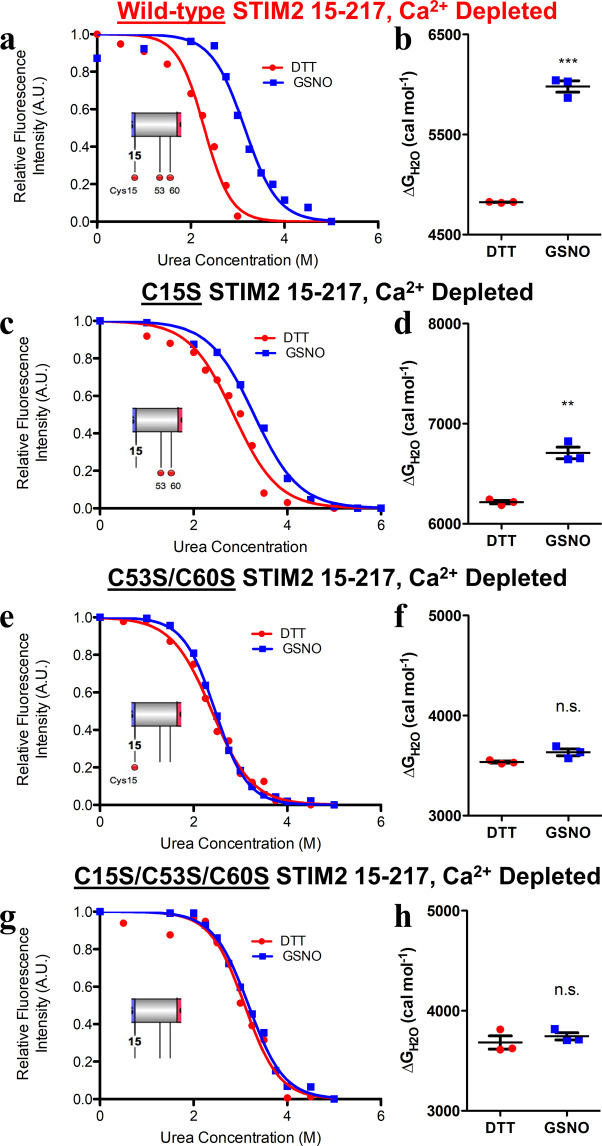
Thermodynamic stability of Ca^2+^-depleted mutant STIM2 15–217 proteins under reducing and *S*-nitrosylating conditions. (**a, b**) Wild-type datasets for comparison (Reproduced from Fig. 2g,h). **(c)** Representative equilibrium denaturation curve of Ca^2+^-depleted (−Ca^2+^) Cys15Ser STIM2 15–217 acquired in the presence of DTT or GSNO. **(d)** Comparison of Δ${{\rm{G}}}_{{{\rm{H}}}_{2}{\rm{O}}}$ extracted from the −Ca^2+^ Cys15Ser denaturation curves. **(e)** Representative equilibrium denaturation curve of Ca^2+^-depleted (−Ca^2+^) Cys53Ser/Cys60Ser STIM2 15–217 acquired in the presence of DTT or GSNO. **(f)** Comparison of Δ${{\rm{G}}}_{{{\rm{H}}}_{2}{\rm{O}}}$ extracted from the −Ca^2+^ Cys53Ser/Cys60Ser denaturation curves. **(g)** Representative equilibrium denaturation curve of Ca^2+^-depleted (−Ca^2+^) Cys15Ser/Cys53Ser/Cys60Ser STIM2 15–217 acquired in the presence of DTT or GSNO. **(h)** Comparison of Δ${{\rm{G}}}_{{{\rm{H}}}_{2}{\rm{O}}}$ extracted from the −Ca^2+^ Cys15Ser/Cys53Ser/Cys60Ser denaturation curves. In (*a* – *h*), data were acquired with 5 μM (*i.e*. 0.11 mg mL^−1^) protein at 20 °C and either 1 mM DTT (red datasets) or 1 mM GSNO (blue datasets). In (*a*, *c*, *e* and *g*), insets show relative locations of modifiable Cys (red spheres). Data in (*b*, *d*, *f* and *h*) are reported as means ± SEM of n = 3 separate experiments for each group and were compared using an unpaired Student’s *t*-test. (**p < 0.01 and n.s. denotes not significant compared to the DTT control).

To confirm this apparent dependence of the Cys15 mediated-stabilization on the *S*-nitrosylation of Cys53 and Cys60, we next performed equilibrium chemical denaturation experiments using the Cys15Ser single, Cys53Ser/Cys60Ser double and Cys15Ser/Cys53Ser/Cys60Ser triple mutant proteins. Here, we used the difference in the Δ${{\rm{G}}}_{{{\rm{H}}}_{2}{\rm{O}}}$ (ΔΔ${{\rm{G}}}_{{{\rm{H}}}_{2}{\rm{O}}}$) between *S*-nitrosylated and reduced conditions as indicators of the stability changes and Ca^2+^-depleted conditions, which exhibit larger GSNO-mediated stabilizations. Consistent with the Ca^2+^-loaded thermal stability assessments, the Cys15Ser protein showed a less pronounced thermodynamic stabilization compared to wild type, reflected by a ΔΔ${{\rm{G}}}_{{{\rm{H}}}_{2}{\rm{O}}}$ of ~+0.5 kcal mol^−1^ (Fig. 4a-d**;** Table 2). The Ca^2+^-depleted Cys53Ser/Cys60Ser double mutation largely abrogated any Cys15-mediated stabilization due to *S*-nitrosylation (Fig. 4e,f; Table 2), showing a ΔΔ${{\rm{G}}}_{{{\rm{H}}}_{2}{\rm{O}}}$ of ~+0.1 kcal mol^−1^. As expected, the Ca^2+^ depleted Cys15Ser/Cys53Ser/Cys60Ser triple mutant did not exhibit any *S*-nitrosylation induced changes in Δ${{\rm{G}}}_{{{\rm{H}}}_{2}{\rm{O}}}$ (Fig. 4g,h**;** Table 2).

Collectively, these data demonstrate that *S*-nitrosylation enhances the stability of STIM2 15–217 in a Cys-specific manner, with the greatest stabilization occurring when all native Cys are available for modification. The unique Cys15 residue contributes to stabilization only when the conserved Cys53 and Cys60 residues are available for *S*-nitrosylation, indicating a remarkable synergistic and cooperative interaction.

### GSNO causes structural changes distant from the modifiable Cys residues

**Figure 5:**
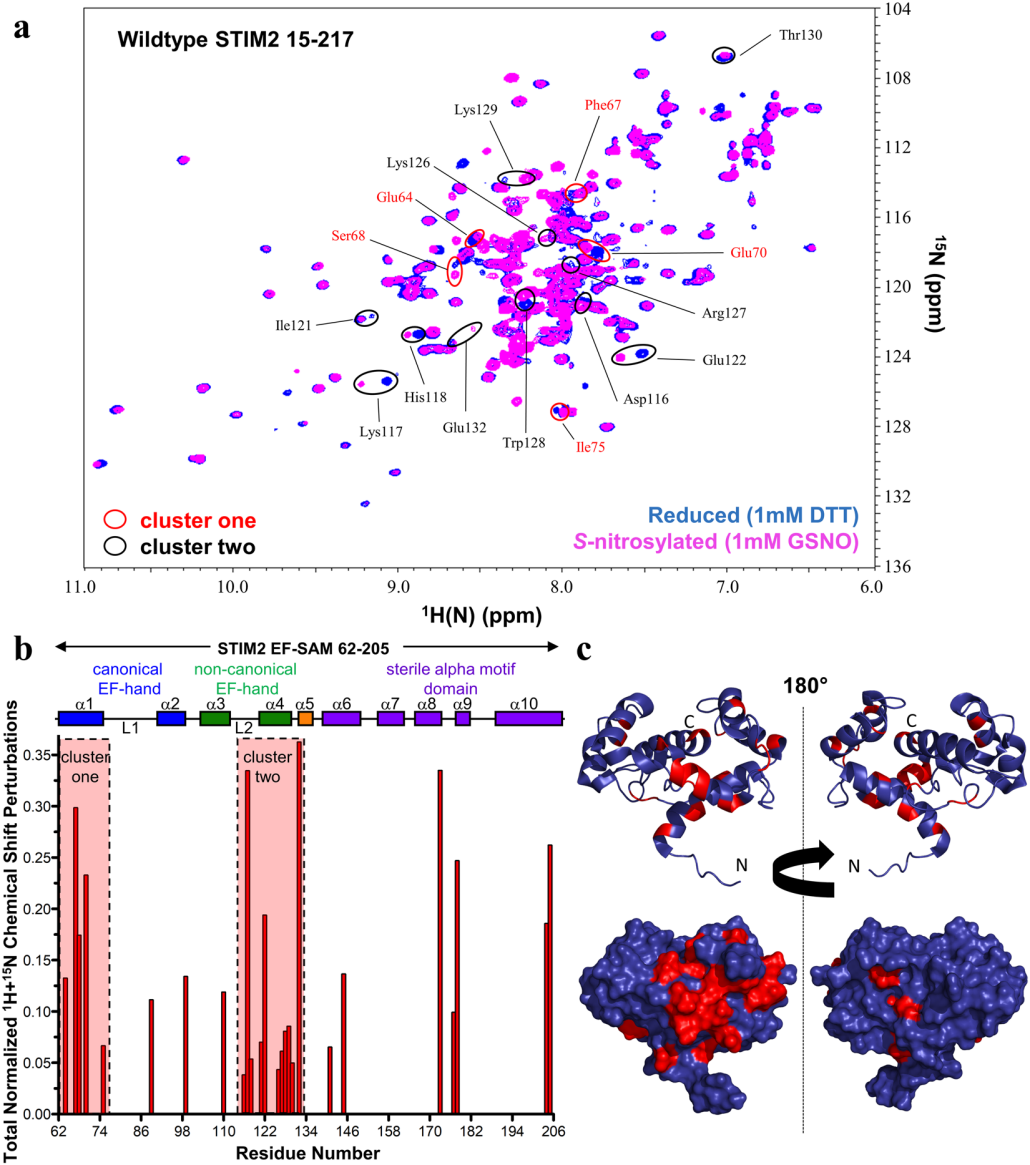
Structural perturbations of wild-type STIM2 15–217 induced by *S*-nitrosylating conditions. (**a**) ^1^H-^15^N HSQC solution NMR spectra of STIM2 15–217 protein under reducing and *S*-nitrosylating conditions. Spectra were acquired at 600 MHz and 20 °C in buffers containing 20 mM Tris, 100 mM NaCl, 10 mM CaCl_2_, pH 7.4 and either 1 mM DTT (blue crosspeaks) or 1 mM GSNO (magenta crosspeaks). **(b)** Total normalized CSPs determined by comparing the chemical shifts extracted from the spectra shown in (*a*). Total^1^H and^15^N CSPs [(ΔH)^2^ + (0.14 × ΔN)^2^]^1/2^ are plotted relative to the STIM2 EF-SAM residue number. The relative location of the helices (rectangles) making up EF-SAM are shown above the plot. Dashed boxes indicate the two largest clusters of CSPs identified relative to sequence space. The H(^15^N) crosspeaks corresponding to the residues and CSPs within these clusters are labeled and bounded by ellipses in (*a*). **(c)** Cartoon representation of the protein backbone trace and surface structure of STIM2 EF-SAM highlighting the location of all CSPs (red) shown in (*b*) relative to three-dimensional space. STIM2 EF-SAM chemical shifts are from BMRB accession code 17289. The image in (*c*) was rendered in PyMOL (v1.7; Schrodinger, LLC) using the 2L5Y.pdb coordinates. Data in (*a* and *b*) are representative of n = 2–3 separate experiments for each condition.

Having uncovered a Cys-dependent synergism in the *S*-nitrosylation-mediated stabilization of STIM2 15–217, we next used solution NMR spectroscopy to probe the structural basis for this phenomenon. In the presence of Ca^2+^ and under reducing conditions (*i.e*. 1 mM DTT), the STIM2 15–217 ^1^H-^15^N-HSQC spectrum exhibited highly dispersed amide ^1^H(^15^N) crosspeaks ranging from ~6.5 to 11 ppm in the ^1^H dimension, suggesting a well-folded conformation. After ultrafiltration exchange from DTT into a GSNO containing buffer, the spectrum showed numerous ^1^H(^15^N) chemical shift perturbations (CSP)s, indicative of structural changes (Fig. 5a). To determine whether the structural changes were localized on the variable N-terminal region containing the three Cys or included more global effects, we compared the ^1^H-^15^N-HSQC spectrum of STIM2 15–217 acquired in the presence of DTT to the previously assigned spectrum of Ca^2+^-loaded STIM2 EF-SAM^17^ (BMRB code 17289). Most ^1^H(^15^N) resonance signals of STIM2 15–217 matched those observed for EF-SAM in isolation, suggesting EF-SAM adopts a very similar conformation in the context of the full luminal domain.

Given these spectral similarities, we calculated the total normalized ^1^H and ^15^N CSP caused by *S*-nitrosylation for each EF-SAM ^1^H(^15^N) resonance identified within the STIM2 15–217 spectra (Fig. 5b). Mapping the CSPs relative to the EF-SAM primary structure revealed two major clusters of residue-specific perturbations: one on the entering helix of the canonical EF-hand (α1) and a second on the exiting helix of the non-canonical EF-hand and the adjacent SAM linking helix (α4 and α5). Mapping the CSPs on the backbone and surface representations of the three-dimensional STIM2 EF-SAM structure (PDB code 2L5Y) revealed that most changes were co-localized on one structural side (Fig. 5c). Cys15, Cys53 and Cys60 are located in the variable N-terminal region outside the STIM2 EF-SAM core; hence, these data suggest that the observed synergism in GSNO-dependent stabilization involves interactions of the variable N-terminal region with a single face of EF-SAM formed by the convergence of α1, α4 and α5. Isolated STIM1 EF-SAM in the presence and absence of GSNO exhibits nearly identical ^1^H-^15^N-HSQC spectra, reinforcing that the structural perturbations observed for STIM2 15–217 are dependent on the presence of the variable N-terminal region containing the Cys residues (Fig. S2).

### GSNO decreases STIM2-mediated basal cytosolic Ca^2+^ levels and SOCE in HEK293T cells

**Figure 6:**
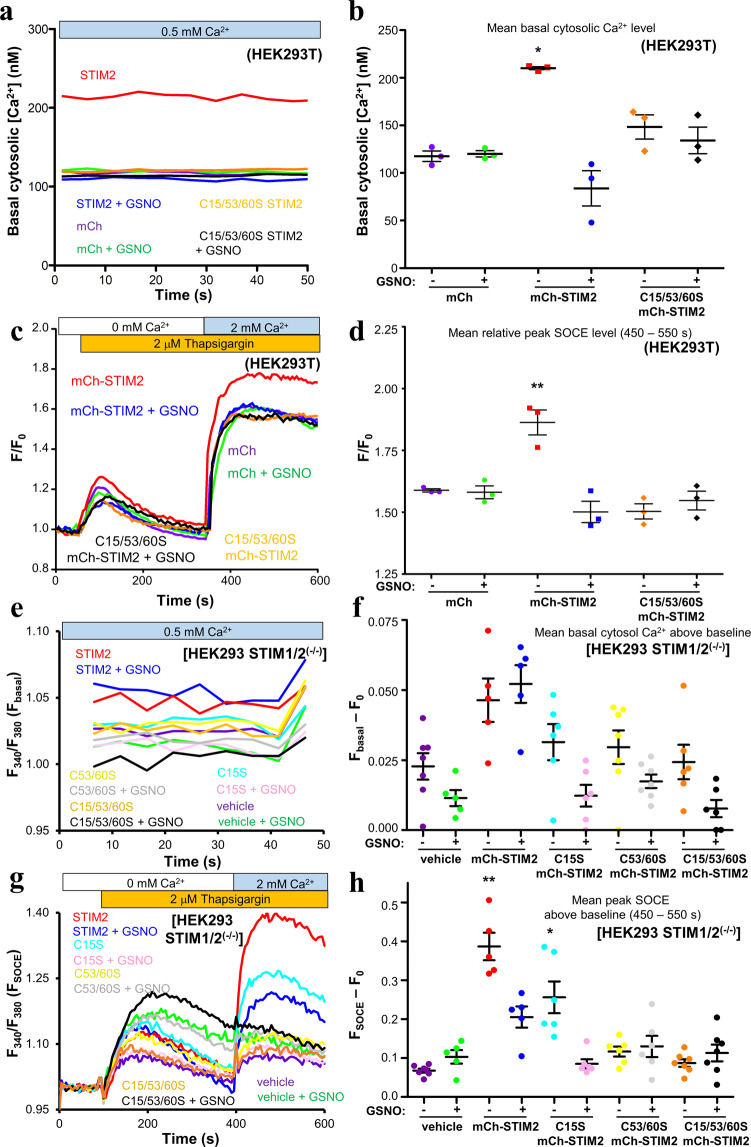
Effects of GSNO on intracellular Ca^2+^ of HEK293T and HEK293 STIM1/2^(−/−)^ cells expressing mCh-STIM2 and eGFP-Orai1. (**a**) Representative resting (basal) cytosolic Ca^2+^ concentration measurements in HEK293T cells calculated from Fura-2 fluorescence ratio (F_340_/F_380_) traces acquired in the presence of 0.5 mM CaCl_2_. **(b)** Comparison of mean basal cytosolic Ca^2+^ concentrations in HEK293T cells. **(c)** Representative peak SOCE responses in HEK293T cells after 2 μM TG and 2 mM net CaCl_2_ addback. Data are shown as relative fold-change over baseline (F/F_0_), where F is F_340_/F_380_ and F_0_ is the mean F_340_/F_380_ measured for 50 s prior to the addition of TG. **(d)** Comparison of the mean peak SOCE level in HEK293T cells after 2 mM CaCl_2_ addback, taken as the mean F/F_0_ between 450–550 s. **(e)** Relative resting basal cytosolic Ca^2+^ concentrations in HEK293 STIM1/2^(−/−)^ cells reported by the mean F_340_/F_380_ traces acquired in the presence of 0.5 mM CaCl_2_. **(f)** Comparison of the relative mean basal cytosolic Ca^2+^ concentrations in HEK293 STIM1/2^(−/−)^ cells, taken as the difference between the mean F_340_/F_380_ ratios measured for 30 s before (F_basal_) and 80 s after the addition of EGTA as the baseline (F_0_). **(g)** Representative peak SOCE responses in HEK293 STIM1/2^(−/−)^ cells after 2 μM TG and 2 mM net CaCl_2_ addback. Data are shown as mean F_340_/F_380_ traces, with F_0_ offset to 1. **(h)** Comparison of the mean peak SOCE levels in HEK293 STIM1/2^(−/−)^ cells, taken as the difference between the mean F_340_/F_380_ ratios between 450–550 s acquired after the 2 mM CaCl_2_ addback (F_SOCE_) and F_0_. In (*b* and *d*), data are means ± SEM of n = 3 separate transfections, and **p < 0.01 and *p < 0.05 denotes significantly different compared to all other groups. In (*f* and *h*), data are means ± SEM of n = 5–7 separate transfections, and **p < 0.01 denotes significantly different compared to all other groups, while *p < 0.05 denotes significantly different compared to all other groups except mCh-STIM2 + GSNO. In (*b*, *d*, *f* and *h*), One-way ANOVA followed by Tukey’s post-*h*oc test was used for the comparisons.

Having established that GSNO causes a Cys-dependent stabilization of the isolated STIM2 luminal region, we next employed Fura-2 ratiometric fluorimetry to ascertain whether the NO donor can modulate full-length STIM2 function *in cellulo*. For these functional experiments, we first measured basal cytosolic Ca^2+^ levels and SOCE in HEK293T cells transiently co-overexpressing enhanced green fluorescent protein tagged Orai1 (eGFP-Orai1) and monomeric cherry fluorescent protein (mCh) tagged STIM2 (mCh-STIM2). Control cells co-overexpressing mCh and eGFP-Orai1 exhibited a mean basal cytosolic Ca^2+^ level of ~118 ± 6 nM. Consistent with past studies^15^, cells co-overexpressing wild-type mCh-STIM2 with eGFP-Orai1 showed an ~2-fold increase basal cytosolic Ca^2+^ compared to the control cells (Fig. 6a,b). Overnight incubation of HEK293T cells co-overexpressing wild-type mCh-STIM2 and eGFP-Orai1 with 250 μM GSNO supplemented into the growth medium muted this basal cytosolic Ca^2+^ enhancement (Fig. 6a,b). To determine whether this GSNO-mediated inhibition was dependent on Cys15, Cys53 and Cys60 of STIM2, similar experiments were performed using a Cys15Ser/Cys53Ser/Cys60Ser triple mutant mCh-STIM2. Unexpectedly, basal cytosolic Ca^2+^ levels of HEK293T cells co-overexpressing the triple mutant mCh-STIM2 protein and eGFP-Orai1 did not significantly differ from control cells co-overexpressing mCh and eGFP-Orai1 either in the presence or absence of GSNO, suggesting that the residue variations at these three sites profoundly affect STIM2 function (Fig. 6a,b).

While STIM2 has been established as a basal Ca^2+^ regulator, it also mediates SOCE^15,35,36^. Thus, we next assessed whether HEK293T cells overexpressing mCh-STIM2 had altered SOCE responses. After extracellular Ca^2+^ chelation with EGTA, thapsigargin (TG) was used to block the sarco/endoplasmic reticulum Ca^2+^ ATPpase pumps. We induced SOCE by adding 2 mM of Ca^2+^ back into the extracellular medium to elicit Ca^2+^ influx through Orai1-composed PM channels. As anticipated, cells overexpressing wild-type mCh-STIM2 and eGFP-Orai1 displayed significantly higher levels of SOCE compared to control cells co-overexpressing mCh and eGFP-Orai1 (Fig. 6c,d). Overnight incubation of wild-type mCh-STIM2 and eGFP-Orai1 expressing cells with 250 μM GSNO abrogated this SOCE enhancement (Fig. 6c,d). Cells co-overexpressing the Cys15Ser/Cys53Ser/Cys60Ser triple mutant mCh-STIM2 and eGFP-Orai1 failed to elicit SOCE above levels observed in the control group either with or without GSNO treatment (Fig. 6c,d). Note that wild-type and mutant mCh-STIM2 expression levels were similar in the presence and absence of GSNO (Fig. S3), as previously observed with mCh-STIM1^27^.

Recent studies suggest that STIM2 may bind and activate STIM1 at ER-PM junctions under resting ER Ca^2+^ conditions, increasing basal cytosolic Ca^2+^ levels^35,37,38^. Thus, we next assessed basal cytosolic Ca^2+^ and SOCE in HEK293 cells with endogenous *STIM1* and *STIM2* deletions [STIM1/2^(−/−)^] induced by CRISPR/Cas9 gene editing^35^. In contrast to HEK293T cells with unaltered endogenous *STIM* genes, eGFP-Orai1 and mCh-STIM2 co-expression did not significantly enhance basal cytosolic Ca^2+^ in the HEK293 STIM1/2^(−/−)^ cells compared to vehicle-transfected controls, and GSNO treatment had no effect on this basal Ca^2+^ (Figs. 6e,f). We similarly assessed the ability of mCh-STIM2 Cys15Ser single, Cys53Ser/Cys60Ser double and Cys15Ser/Cys53Ser/Cys60Ser triple mutant protein to modulate basal cytosolic Ca^2+^ when co-expressed with eGFP-Orai1, finding no differences compared to vehicle-transfected HEK293 STIM1/2^(−/−)^ cells with or without GSNO treatment (Fig. 6e,f).

Next, we used the TG and Ca^2+^ addback approach to assess the SOCE responses. As observed with the HEK293T cells (Fig. 6c,d), co-expression of mCh-STIM2 and eGFP-Orai1 in HEK293 STIM1/2^(−/−)^ cells significantly enhanced peak SOCE compared to vehicle-transfected controls; further, GSNO significantly suppressed wild-type mCh-STIM2-mediated peak SOCE (Fig. 6g,h). Augmented peak SOCE was also induced by mCh-STIM2 Cys15Ser single mutant co-expression with eGFP-Orai1; however, the single mutant-mediated SOCE response was significantly lower than the wild-type mCh-STIM2 expressing cells. GSNO treatment significantly decreased SOCE in HEK293 STIM1/2^(−/−)^ cells expressing the Cys15Ser mutant (Fig. 6g,h). In contrast, the mCh-STIM2 Cys53Ser/Cys60Ser double mutant expressing HEK293 STIM1/2^(−/−)^ cells were unable to induce SOCE, as observed for our Cys15Ser/Cys53Ser/Cys60Ser triple mutant both in the HEK293T and these HEK293 STIM1/2^(−/−)^ cells. GSNO had no effect on the double and triple mutant SOCE responses (Fig. 6g,h).

Collectively, our Fura-2 data suggest that STIM2 can enhance basal cytosolic Ca^2+^ levels and peak SOCE responses in HEK293 cells, and this STIM2 function can be suppressed by GSNO- or mutation-mediated modification of the Cys in the N-terminal region of the protein. Further, while STIM2-induced SOCE can occur independent of STIM1 in HEK293 cells, basal cytosolic Ca^2+^ upregulation requires the presence of STIM1.

## Discussion

The structural and biophysical mechanisms underlying the marked effects of several different luminal domain post-translational modifications on STIM1 Ca^2+^ sensing have been studied including *N*-glycosylation^39^, *S*-glutathionylation^26^, disulfide formation^40^ and *S*-nitrosylation^27,28^. In contrast, the effects of STIM2 luminal domain post-translational modifications are much less understood. Here, we demonstrate that both the basal cytosolic Ca^2+^ and SOCE regulating functions of STIM2^15,36^ are highly sensitive to luminal domain Cys modifications. Further, we reveal that three modifiable Cys found in the luminal region can synergistically contribute to the sensitivity of STIM2 to *S*-nitrosylation, unlike STIM1, which contains only two luminal Cys.

EF-SAM constitutes the highly conserved luminal domains of STIMs that are necessary for maintaining a quiescent state when bound to Ca^2+^ and inducing the conformational changes that activate Orai1 channels after Ca^2+^ depletion^3,19,41^. In the absence of Ca^2+^, STIM2 EF-SAM (residues 62–205) retains a well-folded structure in contrast to the Ca^2+^-depletion induced structural destabilization of STIM1 EF-SAM (residues 58–201)^17,20,21,42,43^. Moreover, previous urea-induced kinetics of unfolding studies indicate that Ca^2+^ loaded STIM1 EF-SAM unfolds>3-times faster than STIM2 EF-SAM^44^. In agreement with these phenomena, we found that under reducing conditions, the full luminal domain of STIM2 (residues 15–217) displays a larger Δ${{\rm{G}}}_{{{\rm{H}}}_{2}{\rm{O}}}$ compared to the full luminal domain of STIM1 (residues 23–213). Specifically, in the Ca^2+^ loaded state, the STIM2 15–217 Δ${{\rm{G}}}_{{{\rm{H}}}_{2}{\rm{O}}}$ is ~+1.0 kcal mol^−1^ larger than STIM1 23–213 (*i.e*. Δ${{\rm{G}}}_{{{\rm{H}}}_{2}{\rm{O}},{\rm{STIM}}2,{\rm{DTT}}}$ − Δ${{\rm{G}}}_{{{\rm{H}}}_{2}{\rm{O}},{\rm{STIM}}1,{\rm{DTT}}}$ = +1.0 kcal mol^−1^), and in the Ca^2+^ depleted state, Δ${{\rm{G}}}_{{{\rm{H}}}_{2}{\rm{O}},{\rm{STIM}}2,{\rm{DTT}}}$ − Δ${{\rm{G}}}_{{{\rm{H}}}_{2}{\rm{O}},{\rm{STIM}}1,{\rm{DTT}}}$ is ~+3.0 kcal mol^−1^ (Table 2). Exchange into GSNO significantly stabilizes the luminal domain of both STIM paralogs; however, Δ${{\rm{G}}}_{{{\rm{H}}}_{2}{\rm{O}}}$ becomes smaller for STIM2 15–217 compared to STIM1 23–213 in the Ca^2+^-loaded state (Δ${{\rm{G}}}_{{{\rm{H}}}_{2}{\rm{O}},{\rm{STIM}}2,{\rm{GSNO}}}$ − Δ${{\rm{G}}}_{{{\rm{H}}}_{2}{\rm{O}},{\rm{STIM}}1,{\rm{GSNO}}}$ = −0.4 kcal mol^−1^) and remains larger for STIM2 15–217 compared to STIM1 23–213 in the Ca^2+^-depleted state (Δ${{\rm{G}}}_{{{\rm{H}}}_{2}{\rm{O}},{\rm{STIM}}2,{\rm{GSNO}}}$ − Δ${{\rm{G}}}_{{{\rm{H}}}_{2}{\rm{O}},{\rm{STIM}}1,{\rm{GSNO}}}$ = +2.8 kcal mol^−1^) states (Table 2). In other words, under *S*-nitrosylating conditions, STIM1 23–213 is more stable than STIM2 15–217 in the presence of Ca^2+^, whereas STIM2 15–217 is more stable than STIM1 23–213 in the absence of Ca^2+^. The switch in stability rank order occurs because *S*-nitrosylation affects STIM1 and STIM2 luminal domains in disparate manners under Ca^2+^-replete (ΔΔ${{\rm{G}}}_{{{\rm{H}}}_{2}{\rm{O}},{\rm{STIM}}1}$ = +2.0 kcal mol^−1^ versus ΔΔ${{\rm{G}}}_{{{\rm{H}}}_{2}{\rm{O}},{\rm{STIM}}2}$ = +0.6 kcal mol^−1^) and -deplete (ΔΔ${{\rm{G}}}_{{{\rm{H}}}_{2}{\rm{O}},{\rm{STIM}}1}$ = +1.5 kcal mol^−1^ versus ΔΔ${{\rm{G}}}_{{{\rm{H}}}_{2}{\rm{O}},{\rm{STIM}}2}$ = +1.2 kcal mol^−1^) conditions (Table 2). Thus, the non-conserved N-terminal region of STIM2 indeed stabilizes the EF-SAM core and GSNO enhances this stabilization, but the degree of the GSNO-mediated stabilization is different for STIM2 15–217 compared to the equivalent STIM1 23–213 region, with *S*-nitrosylation more robustly reinforcing the brake on STIM1 activation in the Ca^2+^-loaded state and the brake on STIM2 activation in the Ca^2+^-depleted state.

Human STIM2 contains three modifiable Cys in the non-conserved luminal region: Cys15, Cys53 and Cys60. Simultaneous mutation of these three Cys to Ser abrogated the GSNO-mediated stabilization observed with the wild-type protein (Figs. 3 and 4). Similarly, the Cys53Ser/Cys60Ser double mutant almost completely blunted STIM2 15–217 stabilization by GSNO. STIM2 Cys53 and Cys60 are conserved in sequence with Cys49 and Cys56 of STIM1 (Fig. 1), which were also found to mitigate GSNO-mediated stabilization of STIM1 23–213 when mutated to Ser^27^. Despite the location at the N-terminus, we found that mutating the Cys unique to STIM2 (Cys15Ser) also significantly attenuated the GSNO-mediated STIM2 15–217 stabilization; however, the remaining modifiable Cys53 and Cys60 residues were able to promote an ~+0.5 kcal mol^−1^ stabilization (Table 2). In contrast, when only the unique Cys15 was available for modification in the Cys53Ser/Cys60Ser STIM2 15–217 protein, *S*-nitrosylating conditions failed to thermodynamically stabilize STIM2 15–217 (Table 2). Collectively, these observations reveal an extraordinary Cys-dependent synergy in GSNO-mediated stabilization of STIM2 15–217, where any contribution by Cys15 is dependent on the preserved *S*-nitrosylability and native conformation endowed by non-variant Cys53 and Cys60 positions.

*S*-Nitrosylation can profoundly affect the structure of proteins. Recently, CD and NMR spectroscopy revealed that *S*-nitrosylation of galectin-2 at Cys57 increases thermal stability by ~+5 °C concomitant with structural alterations that prevent oxidation-induced destabilization^45^. Likewise, we utilized solution NMR spectroscopy to characterize the structural changes associated with *S*-nitrosylation of STIM2 15–217. Our NMR data revealed that numerous ^1^H(^15^N) peaks undergo large CSPs due to alterations in chemical environment (Fig. 5). Not only were unassigned peaks of the variable N-terminal region affected, but numerous previously assigned ^1^H(^15^N) peaks^17^ that cluster on one face of the EF-SAM core were also affected. A control NMR titration of GSNO into an isolated STIM1 EF-SAM sample showed no CSPs (Fig. S2). Thus, these structural data suggest that the three *S*-nitrosylated Cys sulfhydryls may be looping around to interact with the EF-SAM core. *S*-Nitrosylation may be either mediating new interactions or changing existing interactions between these regions in some fashion to facilitate the enhanced stability. Remarkably, the interface identified in the present study by CSP mapping using the intact full STIM2 luminal region is analogous to the STIM1 N-terminal region:EF-SAM interface identified by titrating N-terminal peptides into a solution of EF-SAM^27^. Further, the same N-terminal region:EF-SAM interface has also been recently identified in a crystal structure of the full *C. elegans* luminal domain^42^. Mechanistically, we identified the converging region of α1, α4 and α5 as an important contact site for the N-terminal region. The α1 and α4 helices have been identified as undergoing extensive unfolding in response to Ca^2+^ depletion in the *C. elegans* structure^42^. Thus, we posit that the STIM2 variable N-terminal region interaction with α1, α4 and α5 stabilizes the folded state of these helices, inhibiting the destabilizing conformational change required for activation.

Consistent with our *in vitro* assessments showing enhanced luminal domain stability under *S*-nitrosylating conditions, elevated basal cytosolic Ca^2+^ and SOCE-driven cytosolic Ca^2+^ concentrations in HEK293T cells caused by mCh-STIM2 over-expression were suppressed by exposure to GSNO (Fig. 6). Further, the lower level of SOCE induced by the Cys15Ser mutant and susceptibility to GSNO-mediated inhibition is in-line with the mutation-induced stabilization compared to wild-type and GSNO-mediated stabilization we observed in our chemical denaturation experiments (Table 2). In contrast, the thermodynamic stability of STIM2 15–217 Cys53Ser/Cys60Ser and Cys15Ser/Cys53Ser/Cys60Ser was not significantly different from one another, both exhibiting decreased stability compared to wild-type in the absence of Ca^2+^ (Table 2). Despite this luminal domain destabilization, both the double and triple mutations caused a loss of function in our HEK293 cell experiments (Fig. 6). Accordingly, GSNO treatment of cells expressing these double and triple mutants also had no effect.

Several explanations may underlie the loss of function observed for the double and triple mutant mCh-STIM2. Similar conformations may be induced by these mutation sets that prevent the specific protein interactions necessary for STIM2 activation. This notion is supported by our data showing the Cys53Ser/Cys60Ser and Cys15Ser/Cys53Ser/Cys60Ser mutations both destabilized the Ca^2+^-depleted state by ~−1.3–−1.4 kcal mol^−1^, despite the single Cys15Ser mutant increasing the stability by ~+1.4 kcal mol^−1^ (Table 2). Further, we acquired an ^1^H-^15^N-HSQC of Ca^2+^ loaded, ^15^N-labeled STIM2 15–217 Cys15Ser/Cys53Ser/Cys60Ser, finding that the ^1^H(^15^N) peaks were largely undetectable and severely broadened (not shown). The degenerate NMR spectrum could be caused by conformational exchange (*i.e*. multiple conformations) and/or higher order protein assembly, which are both indicative of some fundamental structural change. Additionally, previous studies showed that both Ca^2+^ binding affinity and EF-SAM stability are integral to dictating the distinct functions of human STIM1 and STIM2^15,17^. Hence, changes in Ca^2+^ binding affinity may underlie the inhibited function. New interactions between the mutated STIM2 luminal domain and the N-terminal mCh or other endogenous proteins may cause the loss of function. Interestingly, the Cys49Ala/Cys56Ala STIM1 double mutant was shown to have a severely compromised ability to induce SOCE in mouse embryonic fibroblast with endogenous STIM1 knockout^40^. This phenotype was in contrast to Cys49Ala/Cys56Ala STIM1 expression in DT40 STIM1 knockout cells, which induced constitutive Ca^2+^ entry^26^. Thus, cell/environment-specific differences may contribute to inhibition versus activation phenotypes. Nevertheless, our *in cellulo* data unequivocally demonstrate an extraordinary sensitivity in STIM2 function to modifications of the Cys residues within the luminal region. This sensitivity is consistent with work showing that swapping the STIM2 N-terminal region (residues 15–69) with the STIM1 N-terminal region (residues 23–66) leads to a switch in the function of the resulting full-length STIM2 chimera, which enhances resting Ca^2+^ and SOCE compared to wild-type STIM2^18^.

*In vivo*, STIM2 isoforms have been identified with long 87 residue and short 14 residue signal peptides (Fig. 1a). The N-terminal Cys is located immediately downstream of the cleavage site in both isoforms (*i.e*. Cys15 versus Cys88); thus, it is interesting to speculate that *S*-nitrosylation of Cys15 (Cys88 in the long isoforms) could add another layer of regulation to STIM2 by modulating ER targeting and processing efficiency. Once in the ER, STIM2 is an integral regulator of basal cytosolic Ca^2+^ and SOCE^15,36^, as was reinforced in the present study. With replete ER Ca^2+^ stores, active STIM2 at ER-PM junctions trap STIM1 at the same sites, enhance the coupling of STIM1 with Orai1 and augment cytosolic Ca^2+^ entry^37,38^. Consistent with this mechanism, we found that mCh-STIM2 co-expression with eGFP-Orai1 in HEK293 STIM1/2^(−/−)^ cells did not enhance basal cytosolic Ca^2+^, in contrast to HEK293T cells with intact endogenous *STIM* genes, which showed enhanced basal cytosolic Ca^2+^ (Fig. 6). However, expression of mCh-STIM2 in HEK293 STIM1/2^(−/−)^ cells increased peak SOCE, indicating that STIM2 can regulate SOCE independent of STIM1 in this cell type.

**Figure 7:**
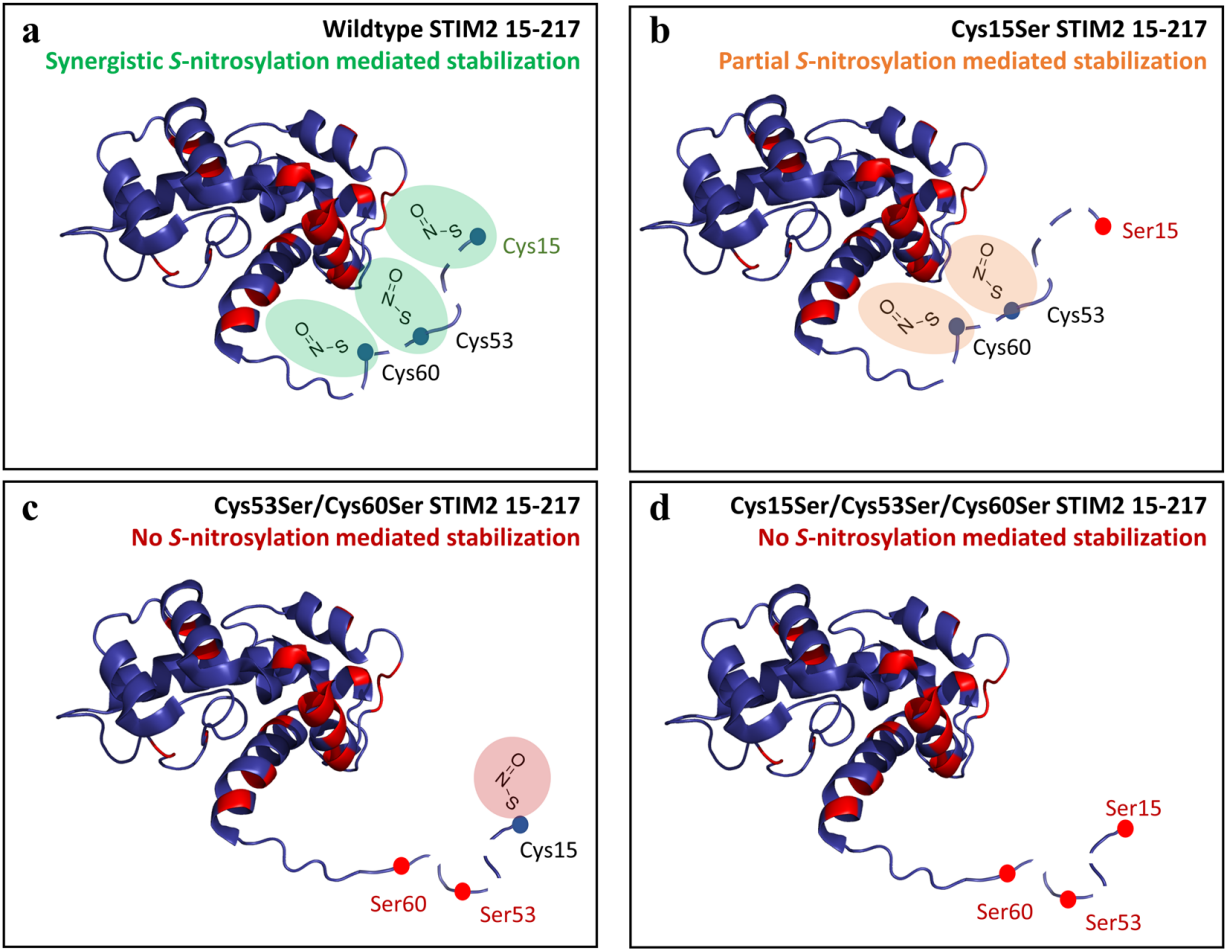
Proposed structural mechanism of *S*-nitrosylation mediated synergistic stabilization of the STIM2 15–217 luminal domain. (**a**) *S*-Nitrosylation of Cys15, Cys53, and Cys60 within the variable N-terminal region of STIM2 results in new or altered contacts with one side of the EF-SAM core, primarily clustered at the convergence point of α1, α4 and α5. **(b)** Blocking *S*-nitrosylation of Cys15 via mutation (Cys15Ser) abolishes >50% of the enhanced stability. (**c**) Blocking *S*-nitrosylation at the Cys53 and Cys60 sites via mutation (Cys53Ser/Cys60Ser) but retaining the ability for Cys15 *S*-nitrosylation eliminates GSNO-mediated stabilization. (**d**) Blocking *S*-nitrosylation at all luminal Cys via mutation (Cys15Ser/Cys53Ser/Cys60Ser) similarly eliminates GSNO-mediated stabilization. Thus, the contribution of Cys15 to *S*-nitrosylation-mediated stabilization is contingent on a preserved *S*-nitrosylability and conformation endowed by non-variant Cys53 and Cys60 positions. In (*a* – *d*), the images showing the backbone structures (blue cartoon) and the contacts (red) were rendered in PyMOL (v1.7; Schrodinger, LLC) using the 2L5Y.pdb coordinates and the CSPs shown in *Fig*. *5*.

Increased STIM2 expression has been linked to numerous diseases, independent of STIM1 (reviewed in^46^). For example, within the vascular system, STIM2 (but not STIM1) protein expression is upregulated in pulmonary arterial smooth muscle cells from patients with pulmonary arterial hypertension^47^. Interestingly, ≥sub-μM concentrations of NO donors have been shown to indirectly inhibit SOCE in platelets through the activation cGMP dependent protein kinase and the possible phosphorylation of STIM2^48^. Biophysically, our data reveal that *S*-nitrosylation stabilizes the full luminal domain of STIM2 in a Cys-specific manner, whereby a unique Cys residue present in the Ca^2+^ sensing region of STIM2 (but not STIM1) is involved in a synergistic stabilization. Structurally, *S*-nitrosylation induces changes to the full luminal domain of STIM2, which are predominantly localized to one face of the EF-SAM core that is involved in destabilization-induced activation (Fig. 7). Functionally, GSNO treatment of HEK293T cells expressing wild-type mCh-STIM2 and eGFP-Orai1 attenuate basal cytosolic Ca^2+^ and SOCE. Thus, our work reveals that NO donors can directly modulate STIM2 Ca^2+^ sensing function through modifications of Cys residues within the luminal domain, which have a potential to profoundly influence physiological and pathophysiological outcomes.

## Methods

### Generation and recombinant expression of full luminal domain STIM2

The full luminal domain of *Homo sapiens* STIM2 (NCBI accession NP_065911.3) corresponding to residues 15–217 was cloned into a pET-28a vector (Novagen) using NheI and XhoI restriction sites and expressed with an N-terminal hexahistidine tag. Cys15Ser, Cys53Ser, and Cys60Ser single-, Cys53Ser/Cys60Ser double- and Cys15Ser/Cys53Ser/Cys60Ser triple-mutants were introduced into separate plasmids by PCR-mediated mutagenesis. Wild-type and mutant proteins were expressed in BL21(DE3) codon plus *E. coli* cells and purified under denaturing conditions as described in the nickel-nitrilotriacetic acid agarose beads manufacturer protocol (HisPur; Thermo Fischer Scientific). Protein was refolded by overnight dialysis in ~65 volumes of 20 mM Tris-HCl, 300 mM NaCl, 1 mM DTT, 5 mM CaCl_2_, pH 8. The N-terminal hexahistidine tag was removed through overnight incubation with ~2 units of bovine thrombin (Calbiochem) per mg of protein. Proteins were further purified via size-exclusion-chromatography using a Superdex 200 10/300 GL (GE Healthcare). Concentrations of each protein were estimated using UV extinction coefficients at 280 nm (ε_280 nm_). The extinction coefficients were ε_280 nm_ = 1.2944 mg mL^−1^ cm^−1^ for wild-type STIM2 15–217, ε_280 nm_ = 1.2953 mg mL^−1^ cm^−1^ for Cys15Ser, Cys53Ser or Cys60Ser single mutant STIM2 15–217, ε_280 nm_ = 1.2961 mg mL^−1^ cm^−1^ for Cys53Ser/Cys60Ser double mutant STIM2 15–217, and ε_280 nm_ = 1.2970 mg mL^−1^ cm^−1^ for Cys15Ser/Cys53Ser/Cys60Ser triple mutant STIM2 15–217 proteins. STIM1 EF-SAM was expressed and purified as recently described^27^.

### Ca^2+^ depletion and S-nitrosylation

The Ca^2+^ depletion condition was achieved by overnight incubation with 50 mM EDTA followed by a 20 × 20 × 20 × 20-fold exchange by ultrafiltration into nominally Ca^2+^ free buffer. GSNO was prepared as described previously^49^, and the concentration was estimated using ε_335 nm_ = 0.92 mM^−1^ cm^−1^^50^. Proteins were exchanged into a buffer containing 1 mM GSNO by ultrafiltration using a 20 × 20 × 20 × 20-fold total buffer exchange.

### Thermal stability by far-UV circular dichroism (CD) spectroscopy

Data were acquired on a Jasco J-815 CD Spectrometer (Jasco Inc.) equipped with a PTC-423S temperature controller using a 0.1 cm path length quartz cuvette in 1 nm increments (20 nm min^−1^), an 8 second averaging time and 1 nm bandwidth. Thermal melts were acquired by monitoring the change in negative ellipticity (mdeg) at the 225 nm peak minima as a function of temperature using a 1 °C min^−1^ scan rate. The apparent midpoints of temperature denaturation (T_m_) were extracted from the thermal melts using Boltzmann sigmoidal fitted curves in GraphPad Prism.

### Equilibrium chemical denaturation curves

Protein samples were diluted to 5 μM and were incubated overnight in the presence of 0–6 M urea at 25 °C. A Cary Eclipse spectrofluorimeter (Varian/Agilent Inc.) was used to detect intrinsic fluorescence intensity for each sample using an excitation wavelength (λ_ex_) = 280 nm and emission wavelength (λ_em_) =339 or 337 nm for the Ca^2+^-loaded and -depleted conditions, respectively. Protein thermodynamic stability parameters were extracted from the equilibrium chemical denaturation curves using a two-state unfolding model and the linear extrapolation method^34,51^.

### Solution NMR spectroscopy

^1^H-^15^N HSQC spectra of STIM2 15–217 were acquired on a Varian/Inova 600 MHz NMR spectrometer equipped with a triple resonance HCN cryogenic probe using 48 or 256 transients, 64 increments in the nitrogen dimension, 8,000 Hz ^1^H sweep width, and 2,000 Hz ^15^N sweep width at 20 °C. All NMR samples contained 60 μM 4,4-dimethyl-4-silapentane-1-sulfonic acid and 10% D_2_O (v/v). *S*-Nitrosylated and reduced spectra were compared using two approaches: first, by acquiring spectra on two samples separately containing 1 mM GSNO or 1 mM DTT, and second, by acquiring a spectrum on one sample containing 1 mM GSNO, and re-acquiring a spectrum on the same sample after supplementing with 15 mM DTT. ^1^H−^15^N HSQC spectra of STIM1 EF-SAM were similarly compared using the two approaches; however, the NMR data was acquired using 32 transients, 64 increments in the nitrogen dimension, 8,000 Hz ^1^H sweep width, and 1,800 Hz ^15^N sweep width at 20 °C.

### Mammalian cell culture

HEK293T cells were seeded in 35 mm dishes while HEK293 STIM1/2^(−/−)^ cells (a gift from M. Trebak^35^) were seeded in a 96-well plate. All cells were cultured in Dulbecco’s modified Eagle’s medium (Wisent) supplemented with 10% (v/v) fetal bovine serum (Sigma) and 100 μg mL^−1^ penicillin-streptomycin (Wisent) at 37 °C and 5% CO_2_ in an air humidified incubator. pCMV6 vectors containing wild-type or mutant mCh-STIM2 variants were co-transfected with eGFP-Orai1 into cells at ~70–80% confluency using PolyJet transfection reagent (SignaGen Laboratories) according to the manufacturer’s protocol. The pCMV6-eGFP-Orai1 vector has enhanced green fluorescent protein (residues 1–239; NCBI accession AAB02572.1) fused in-frame to the N-terminus of human Orai1 (residues 2–301; NCBI accession NP_116179.2), interceded by a Gly-Thr linker. The pCMV6-mCh-STIM2 vector was previously described^17^. From the N- to C-terminus, the construct contains the human N-terminal STIM1 signal peptide (residues 1–34; NCBI accession Q13586.3) fused in frame to mCherry (PCRed from mCherry-pRSET-B^52^), a Gly-Thr linker and human STIM2 (residues 15–746; NCBI accession NP_065911.3). To promote *S*-nitrosylation *in cellulo*, the growth medium of select wells/dishes was supplemented with 250 μM GSNO at 8 h post-transfection and incubation was continued overnight. All plasmid DNA was suspended in water (vehicle).

### Fura-2 fluorimetry

HEK293T cells were lifted from 35 mm dishes by pipetting and subsequently incubated with 2 µM Fura-2-AM (Alfa Aesar) for 45 minutes in the dark at 37 °C. Cells were washed twice with HEPES-buffered saline solution (HBSS; 140 mM NaCl, 4.7 mM KCl, 1.13 mM MgCl, 10 mM glucose, and 10 mM HEPES, pH 7.4) before being resuspended in 1.2 mL HBSS buffer containing 0.5 mM CaCl_2_. The cell suspensions were equilibrated for 10 minutes at 22.5 °C before fluorescence assessments using a Cary Eclipse spectrofluorimeter (Varian/Agilent). Fluorescence emission at 510 nm was measured at alternating excitation wavelengths of 340 and 380 nm (F_340_ and F_380_, respectively) in 1 second intervals. After the initial basal Fura-2 F_340_/F_380_ ratios were determined, 1.0 mM EGTA (0.5 mM molar excess over Ca^2+^) was added to the cell suspension. Subsequently, 2 µM TG and 2.5 mM CaCl_2_ (2.0 mM excess over EGTA) was sequentially added to the extracellular solution to gauge SOCE. The data are plotted as a normalized F/F_0_ ratio, where F is the F_340_/F_380_ ratio, and F_0_ is the average F of the first 10 data points (50 s) before the addition of TG. Basal cytosolic Ca^2+^ concentrations were calculated from F as previously described^53,54^ using a Ca^2+^ equilibrium dissociation constant of 225 nM. The Ca^2+^ saturated and depleted conditions were calibrated by adding 0.2% (v/v) Triton X-100 and 10 mM EGTA to the cell suspensions, respectively. The same calibration values were applied to all experiments.

HEK293 STIM1/2^(−/−)^ cells in a 96-well plate were incubated with 1 µM Fura-2-AM for 45 minutes in the dark at 37 °C and subsequently washed twice with 200 μL HBSS before being covered in 100 µL HBSS containing 0.5 mM CaCl_2_. The adherent cells were equilibrated for 10 minutes at ambient temperature before fluorescence assessments using a FlexStation 3 Multi-Mode microplate reader (Medical Expo). Fluorescence emission was measured at alternating excitation wavelengths in 1 second intervals. The FlexStation automated pipettor was used to sequentially deliver 1.0 mM EGTA, 2 μM TG and 2.5 mM CaCl_2_ to the wells. The data are plotted as F_340_/F_380_ ratios, where F_340_ and F_380_ are the 510 nm fluorescence emission intensities at excitation wavelengths of 340 and 380 nm, respectively. The baseline (F_0_) was taken as the mean F_340_/F_380_ ratios measured for 80 s before the addition of TG and in the presence of EGTA. Relative basal cytosolic Ca^2+^ levels were calculated as the difference between the mean F_340_/F_380_ ratios measured for 30 s before the addition of EGTA (F_basal_) and baseline (F_basal_-F_0_). Relative peak SOCE was taken as the difference between the mean F_340_/F_380_ ratios after the 2.5 mM CaCl_2_ addback (F_SOCE_) and baseline (F_SOCE_-F_0_).

### Statistical analysis

An unpaired Student’s *t* test was used to compare two independent groups, and a one-way analysis of variance (ANOVA) followed by Tukey’s post hoc test was used to compare three or more groups. All data are reported as means ± SEM.

## Supplementary information

Supplementary Information.
