# Supplementary material for: Synergistic stabilization by nitrosoglutathione-induced thiol modifications in the stromal interaction molecule-2 luminal domain suppresses basal and store operated calcium entry
Source: Sci Rep. 2020 Jun 23;10:10177. doi: 10.1038/s41598-020-66961-3 (PMC7311479; doi:10.1038/s41598-020-66961-3)
Supplement: Supplementary file 1 — Supplementary Information. [file 41598_2020_66961_MOESM1_ESM.pdf]

Supplementary Information

Title

Synergistic stabilization by nitrosoglutathione-induced thiol modifications in the stromal interaction molecule-2 luminal domain suppresses basal and store operated calcium entry.

Authors

Matthew J. Novello[1], Jinhui Zhu[1,2], MengQi Zhang[1,3], Qingping Feng[1,4] and Peter B. Stathopulos[1,4].

Affiliations

[1]Department of Physiology and Pharmacology, Schulich School of Medicine and Dentistry, the University of Western Ontario, London, Ontario, Canada. N6A5C1.

[2]Present Address: Dentistry, Schulich School of Medicine and Dentistry, the University of Western Ontario, London, Ontario, Canada. N6A5C1.

[3]Present Address: Faculty of Medicine, University of Ottawa, Ottawa, Ontario, Canada. K1H 8M5.

[4]Address correspondence to Qingping Feng (qfeng@uwo.ca) and Peter B. Stathopulos (pstatho@uwo.ca).

Supplementary Information Figure legends

Supplementary Fig. S1. Thermal stability comparison of Ca^{2+}-loaded wild-type mutant STIM2 15-217 proteins under reducing conditions. Comparison of apparent T_m values extracted from the Ca^{2+}-loaded thermal melts for wild-type and all Cys→Ser mutant STIM2 15-217 proteins acquired under reducing conditions. Data are compiled from *Fig. 2* and *Fig. 3*. Data are means ± SEM of $n = 3$ separate thermal melts for each group. Statistical analysis was performed using a one-way ANOVA followed by a Tukey's multiple comparisons test. $P < 0.05$ was considered statistically significant. Groups with the same letter are not significantly different from one another.

Supplementary Fig. S2. Effects of GSNO on the solution NMR spectra of the core STIM1 EF-SAM (residues 58-201) domain. ^{1}H-^{15}N HSQC spectra of STIM1 EF-SAM acquired in the presence (magenta peaks) and absence (blue peaks) of GSNO are overlaid. Spectra were acquired at 600 MHz and 20 ºC in buffers containing 20 mM Tris, 150 mM NaCl, 5 mM $CaCl_2$, pH 7.4 with 1 mM GSNO or without GSNO. The limited chemical shift differences observed between spectra indicates that GSNO minimally affects the structure of the core STIM1 EF-SAM domain, which is highly homologous with STIM2 EF-SAM (*i.e.* 58% sequence identity and 88% sequence similarity).

Supplementary Fig. S3. Effects of GSNO treatment on eGFP-Orai1 and mCh-STIM2 protein expression levels in HEK293T cells. (a) Maximum eGFP fluorescence emission of cell suspensions used in the Fura-2 intracellular Ca^{2+} assessments. Fluorescence emission ($\lambda_{emission}$) at 510 nm was measured using an excitation wavelength ($\lambda_{excitation}$) set to 510 nm. **(b)** Maximum mCh fluorescence of cell suspensions used in the Fura-2 intracellular Ca^{2+} assessments. Fluorescence was measured using an $\lambda_{excitation} = 565$ nm and $\lambda_{emission} = 610$ nm. Wild-type (WT) mCh-STIM2 data are coloured red and blue and Cys15Ser/Cys53Ser/Cys60Ser triple mutant mCh-STIM2 are coloured orange and black for cells incubated in the absence and presence of GSNO, respectively. Data are means ± SEM of $n = 3$ separate transfections. Statistical comparisons were performed using one-way ANOVA followed by Tukey's post-hoc test. Groups with the same letters are not significantly different from one another.

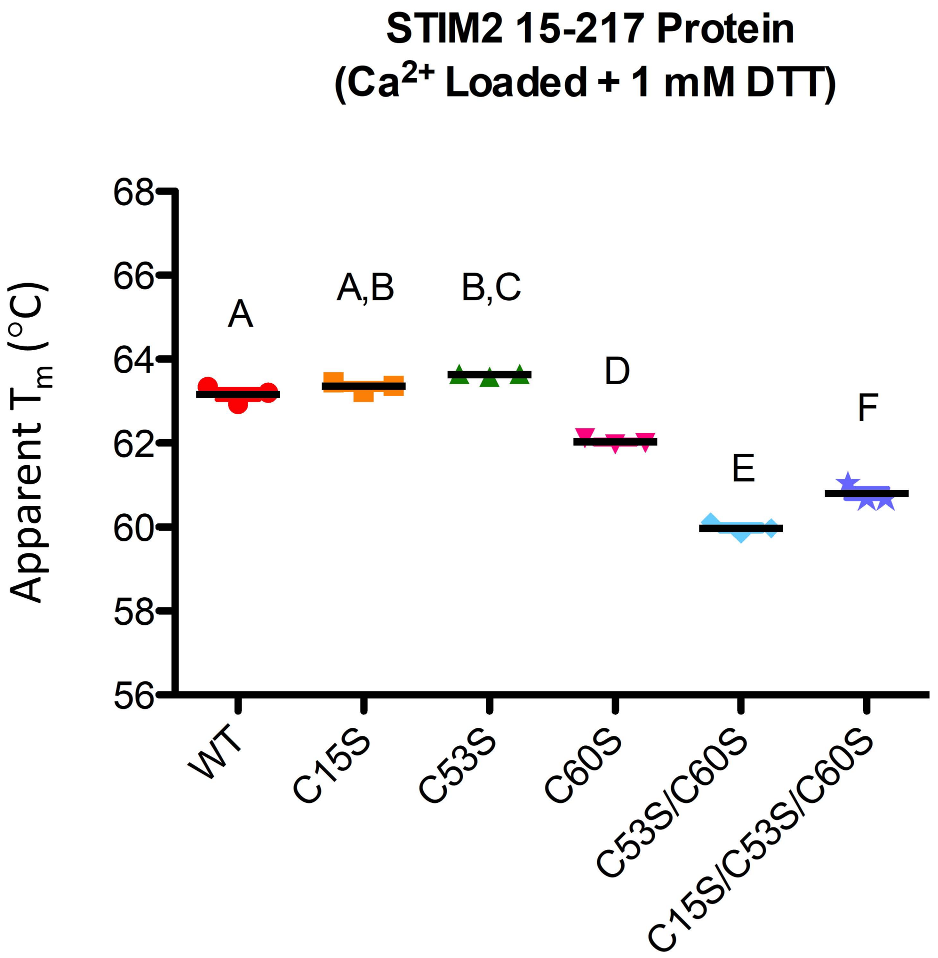

Supplementary Figure S1

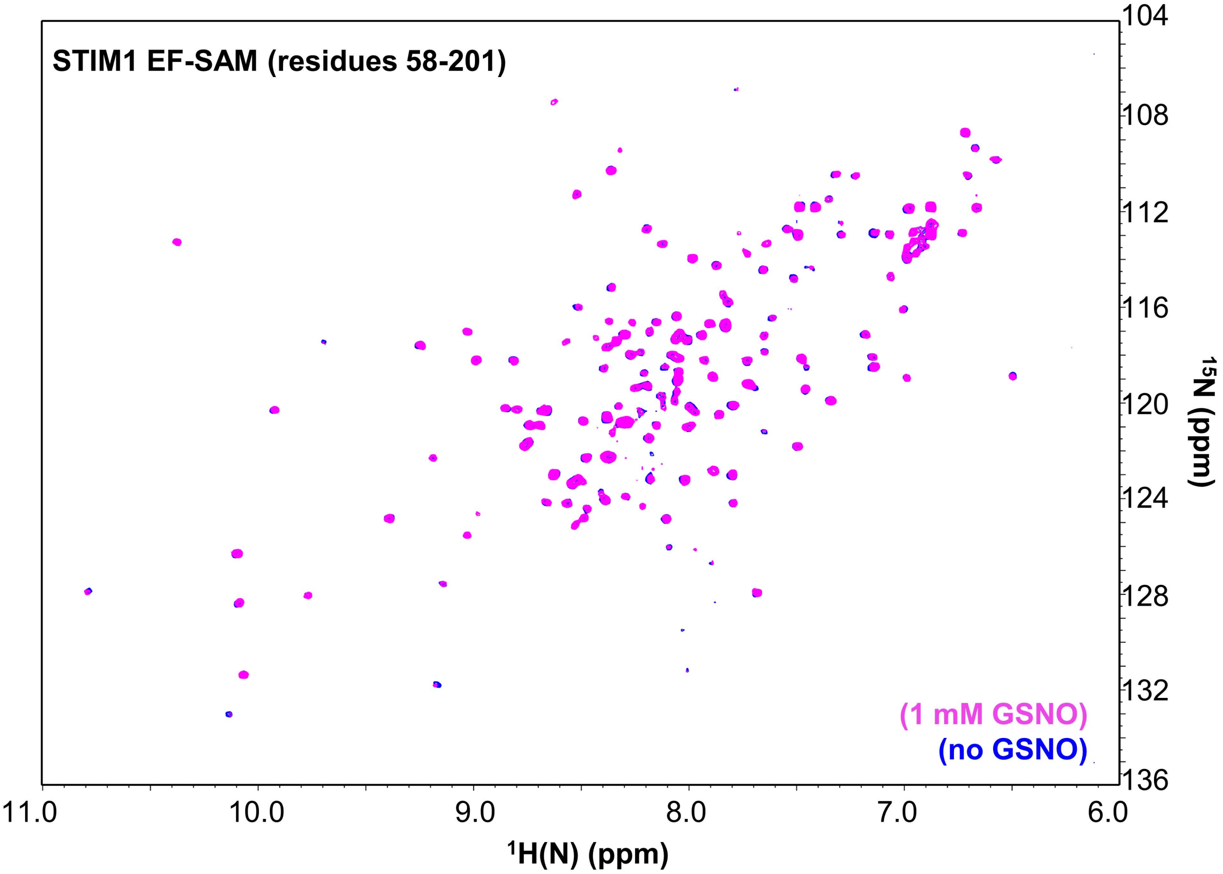

Supplementary Figure S2

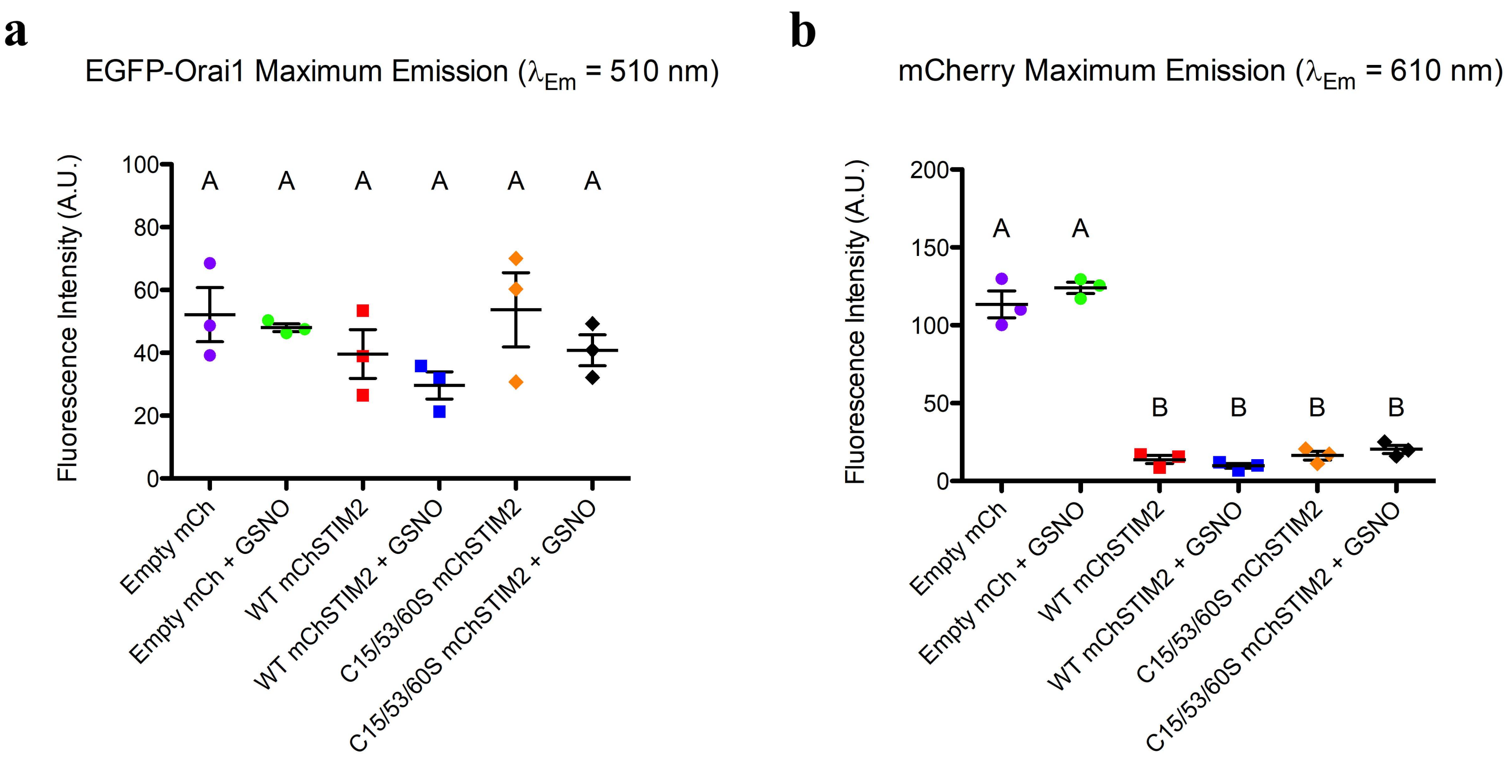

Supplementary Figure S3
